# Why do some Korean parents hesitate to vaccinate their children?

**DOI:** 10.4178/epih.e2019031

**Published:** 2019-07-09

**Authors:** Kyujin Chang, Soon Young Lee

**Affiliations:** Department of Preventive Medicine and Public Health, Ajou University School of Medicine, Suwon, Korea

**Keywords:** Vaccine hesitancy, Vaccination refusal, Health belief model, Korea

## Abstract

**OBJECTIVES:**

Vaccinations for infectious diseases are opposed despite their achievement, and this opposition has recently been revealed in Korea. However, research in Korea has not been vigorous. The authors studied why some Korean parents hesitate to vaccinate their children by applying the health belief model.

**METHODS:**

Parents who hesitate to vaccinate and parents who do not were surveyed in alternative education preschools and elementary schools. They were classified into four types of hesitancy and statistically compared.

**RESULTS:**

Among the 129 subjects, 43 vaccinated without hesitancy, 20 vaccinated on time with hesitancy, 32 vaccinated with a deliberate delay of one month or longer, and 34 did not vaccinate. Vaccination increased with an increase in the awareness that severe outcomes can occur when unvaccinated. Concerns about adverse reactions from vaccinations or direct/indirect experiences affected refusal. Furthermore, perceptions of the lack of meaningfulness of vaccinations, distrust of policy and safety management, influence of leaders or activists in joined organizations, and experts of Korean traditional or alternative medicine affected refusal. Explanations by doctors, text messages and mails from institutions, and concerns about disadvantages caused by not complying with government policies increased vaccination.

**CONCLUSIONS:**

The reasons for vaccine hesitancy and acceptance were similar to the results of international research. Health authorities and professionals should communicate sufficiently and appropriately with hesitant parents and find ways to rationally resolve social conflicts. However, this sample was small and there is little Korean research, so more in-depth and diverse researchs are needed.

## INTRODUCTION

Vaccination against infectious diseases is a modern scientific achievement, and it is recognized as an essential protector for the lives and health of children [1]. However, there has been continuous trends of vaccination refusal in many countries to date from the first smallpox vaccination in the eighteenth century [2,3]. In particular, there are still many anti-vaccination groups in advanced countries, such as the USA, Europe, Australia, and Japan, even though the socioeconomic barriers have been sufficiently eliminated [1,4-6]. Furthermore, researchs on the characteristics of the groups, the socioeconomic effects, mandatory or liberalization policies, and ethics are ongoing [4,5,7-10].

Existing studies often apply the concept of vaccine hesitancy (VH) presented by the World Health Organization (WHO) in regard to parental attitudes and decisions about required child vaccinations. This refers to deliberately delaying or refusing vaccination even though services are accessable [7]. Based on this, many studies make four classifications: First, vaccination without hesitancy; second, vaccination on time with hesitancy; third, deliberately delaying vaccination for one month or longer with hesitancy; and fourth, deliberate vaccination refusal with hesitancy [11,12].

The proportion of required child vaccinations is known to be high in Korea, and VH studies are rarely reported [13,14]. The concept and classifications of VH are also unfamiliar in Korea. However, with “ANAKI” (Korean abbreviation of “raising children without medication”) becoming a societal issue in 2017, movements to refuse modern medicine including vaccines have appeared. Similar to there being parents who hestiate vaccinations or medical treatments in alternative education preschools and schools overseas [15], authors knew that those parents also existed in Korean alternative education facilities. Of course, their scale needs to be identified through a national survey, but there are practical limitations. Therefore, authors first surveyed accessible parents to examine their VH reasons based on the Health Belief Model (HBM).

## MATERIALS AND METHODS

The subjects were recruited from alternative education preschools and elementary schools in which there would presumedly be VH parents. There are 78 locations in the Association of Alternative Education Preschools, among which 26 locations are in Southern Gyeonggi province and 9 are in Northern. Preschools outside association could not be identified. Although 246 locations (59 in Gyeonggi) of alternative education schools are registered in their Association, most are middle or high schools. As there are no elementary school dataset, it was estimated 20 locations by checking website one by one. It was difficult to get a standardized sample. The area in which many are located and which authors could directly visit was Southern Gyeonggi. Twenty-six preschools and 7 elementary schools were contacted in person, by mail, by phone, and by e-mail. The subject selection criteria at the participating facilities were Korean parents with one child or more in second grade in elementary school or younger as of 2018 (i.e., a child born after January 1, 2010). According to the standard schedule of the Korea Centers for Disease Control and Prevention, child vaccinations are completed at the age of 12. Therefore, they were first targeted. However, considering the repeated revision of schedule, only 2 additional vaccinations between the ages of 10 and 12, decreased parental vaccination interest after the child starts elementary school, and the difficulty to remember previous vaccinations, the birth year were limited to 2010 or later. Also, underaged parents, non-Korean or naturalized parents, and caregivers of grandchildren were excluded.

**Figure 1.**
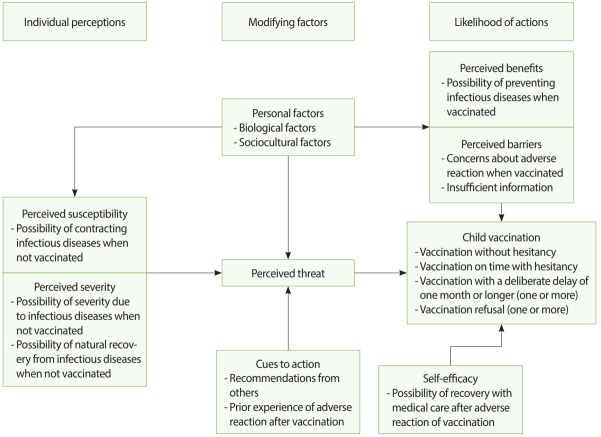
Process of child vaccination decision making according to the Health Belief Model.

The authors applied a structured qustionnaire that was revised based on previous studies. The qustionnaire was comprised of basic human information, common HBM questions, and items relating to the four VH classifications. The HBM questions were structured by referencing the previous studies and covered the following topics: perceived susceptibility and severity, which are individual perceptions; personal factors and cues to action, which are modifying factors; and perceived benefits and barriers, which refer to the possibility of behavior [5,11,16,17]. Previous questions included both interrogative and descriptive sentences, and there were cases of pinpointing a specific vaccination and cases of not. The authors designed the items to be as descriptive as possible so that the subjects could respond whether they agreed or not. The Korean vaccination schedule was presented and the responses to overall vaccination were requested. Additionally, including self-efficacy questions which has been included in the HBM in recent studies [16], the model determining the four VH types were structuralized (Figure 1). Each items reflected VH using the agreement of the responses. Meanwhile, specific questions of the reason for vaccination or hesitation were structured with references [6,18-20]. Items asked the reason for hesitating, delaying, or refusing vaccination; the person who made impacts; and the reason for ultimately vaccinating. Items that fit Korean characteristics, such as Korean traditional medicine, disadvantages due to non-cooperation with government policies, and text messages from institutions, were added. The Korean version questionnaire is attached (Supplementary Material 1). The questionnaires were directly distributed at the facilities, delivered through representatives or teachers, and mailed.

Each participant was given one tricolored pen without other compensation. Statistical analyses were performed using SPSS version 23 (IBM Co., Armonk, NY, USA). The chi-square test for trend test was done to compare groups, and analysis of variance was performed on continuous variables. Additionally, logistic regression analysis was done with HBM factors. The multicollinearity among the variables was identified with the Cramer’s V value. The significance level was 0.05.

### Ethics statement

This study was approved by the Institutional Review Board of Ajou University Hospital on July 24, 2018. The approved informed consent and questionnaire were used.

## RESULTS

Surveys were conducted in 12 preschools and 2 elementary schools between August and November 2018. A total of 141 subjects participated, with 1 to 33 subjects per location. Among those, 1 without children born in 2010 or later, 6 with children with medical illnesses that make vaccinations impossible, 5 with no responses, and 3 with no response on whether the child lives together were excluded. After excluding 12 subjects including duplicates, 129 subjects were finally analyzed.

They were comprised of 43 who vaccinated without hesitancy, 20 who vaccinated on time with hesitancy, 32 who delayed vaccination for one month or longer, and 34 who refused vaccination. There were 119 women whose mean age was 38.1 years old (28-46 years old), among whom 125 were married and 127 lived in Gyeonggi. As for education level, there were 3 high school graduates, 96 college/university graduates, and 31 graduate school graduates. The subjective income levels were upper class for 3 subjects, middle class for 109 subjects, lower class for 14 subjects, and 3 did not respond. The number of children was 1 for 47 subjects, 2 for 69 subjects, and 3 for 13 subjects. The differences among the four groups by item showed no significant results in all items except income level; the vaccination refusal group had more “lower-level” income than the other groups at 23.5% (p=0.006) (Table 1).

Questions on whether the subject saw the danger of infectious diseases and the benefits of vaccination as important were about perceived susceptibility, the first item on perceived severity, the perceived benefits, and self-efficacy. For all four, positive responses were higher with less VH and this trend was statistically significant. The questions about negative disposition toward vaccinations - the first item on perceived barriers, the second item on cues to action, and the second item on perceived severity - showed increasing statistical tendencies for negativity toward vaccinations as the classification became closer to vaccination refusal. Although the second item on perceived barriers and first and third items on cues to action had statistical tendencies, the number of respondents was somewhat lower for the refusal group compared to the deliberately delay group (Table 2).

To identify the correlations among the 10 HBM variables and subjective income levels, which showed statistical differences, Cramer’s V values were calculated. As a result, pairs that exceed 0.5 were perceived susceptibility and the first item of perceived severity (0.595) and perceived susceptibility and perceived benefits (0.579). Pairs that exceed 0.4 were the first item of perceived severity and perceived benefits (0.480) and the first and third items of cues to action (0.411). Cramer’s V values between the other variables were all below 0.4.

A logistic regression analysis using nine HBM items was computed after excluding the perceived susceptibility item based on Cramer’s V value of 0.5. Income levels, which was significant among the socio-demographic variables, was excluded because of large differences in the numbers of subjects in the 3 groups: 3 upper-level, 109 middle-level, and 14 lower-level. For the binary logistic regression, vaccination was recategorized by combining vaccination without hesitancy and vaccination on time with hesitancy and by combining delay and refusal. The chi-square analyses after the combinations still showed significance levels below 0.05. The odds ratios before the adjustment were statistically significant for all items. After the adjustment, the perceived severity was significant (p=0.042) (Table 3).

Apart from HBM questions, all four groups were asked about the perception of the necessity and safety of vaccinations. The result showed statistical tendencies of lower VH being associated with a higher level of perception of necessity (p<0.001) and safety (p<0.001) (Table 2). Sixty-six subjects stated that they refused or deliberately delayed vaccination for one month or longer. After allowing multiple responses, delayed or refused vaccines for Japanese encephalitis were the most common at 41 cases, followed by 34 cases of diphtheria, tetanus toxoid, acellular pertussis (DTaP), 34 cases of measles, mumps, rubella (MMR), 32 cases of inactivated polio vaccine (IPV), 27 cases of chickenpox vaccine, 15 cases of Bacillus Calmette-Guérin (BCG), and 15 cases of hepatitis B vaccine. There were 20 cases of non-vaccination despite not knowing which one.

The hesitant reasons were asked from the group who vaccinated on time with hesitancy, the delay group, and the refusal group. Concerns and experiences of adverse reactions with vaccinations were 60% or more in all groups. The percentage that they distrusted vaccination safety management was around or above 50% as well. The most common reasons for hesitancy by group were as follows: For the group who vaccinated on time with hesitancy, the reasons were in the order of concerns and experiences of adverse reactions, distrust of safety management, and distrust of government policies. For the delay group, the reasons were in the order of concerns and experiences of adverse reactions, distrust of government policies, and distrust of safety management. For the refusal group, the reasons were in the order of concerns and experiences of adverse reactions, distrust of safety management, and the meaninglessness of vaccinations (Table 4). The persons who affected the hesitancy were as follows: For the group who vaccinated on time with hesitancy, the order was online anti-vaccination activists, decision by oneself, and acquaintances. For the delay group, the order was decision by oneself, anti-vaccination activists, acquaintances, and family. For the refusal group, the order was decision by oneself, anti-vaccination activists, acquaintances, activists in joined organizations, and experts of Korean traditional or alternative medicine (Table 5).

The reasons of vaccine acceptance were asked from the group who vaccinated without hesitancy, the group who vaccinated on time with hesitancy, and the group who deliberately delayed vaccination. For the no hesitant group, the reasons were in the order of decision by the parents themselves (72.1%), text messages or mails from medical/health facilities (53.5%), and the explanation of doctors who saw their children (48.8%). For the on-time vaccination group with hesitancy, the reasons were in the order of decision by the parents (85.0%) and concerns about the disadvantages to the children upon failure to cooperate with government policies (30.0%). For the delay group, the reasons were in the order of decision by the parents (59.4%), concerns about the disadvantages (37.5%), and text messages or mails from facilities (28.1%).

## DISCUSSION

Previous studies [11,21,22] reported that the hesitant groups tends to have higher education and income levels. In this study, the proportions of college/university graduation or higher were high at 96.9% and 100.0% for the delay group and refusal group, respectively. However, the proportions were also high at 97.7% and 95.0% for the no hesitant group and the on-time vaccination group with hesitancy, respectively, showing no differences between the groups. Income level was measured via subjective responses rather than quantitative measurements. Although the response for “middle-level” was high for the no hesitant group, the vaccination group with hesitancy, and the delay group at 85.7%, 95.0%, 93.8%, and 79.4% of the refusal group was “middle” and 23.5% was “low,” showing statistical differences. However, only the tendency could be examined because of small sample size, and the reason could not be identified.

The analysis with the HBM showed that the group with lower VH had more concerns about infectious diseases when not vaccinated, which affected the perceived threat and led to vaccination acceptance. In contrast, the more hesitant group thought highly of the possibility of natural recovery even when exposed to infections, which lowered the perceived threat and led to vaccination refusal. Furthermore, lower hesitancy was associated with the perception of benefits that vaccination can prevent infections, having a positive effect to accept vaccination. In contrast, concerns about the adverse reactions from vaccination or not being provided with sufficient information about vaccination appeared to be higher in the hesitant groups and affected refusal. However, insufficient information exceeded 50% in the vaccination groups as well. Cues to action were direct/indirect experiences of adverse reactions. If the level of this factor increased, the perceived threat decreased and affected refusal. For all three relevant items, it was shown that the more hesitant groups had a higher level of this factor and this affected refusal. The self-efficacy item, which was the belief that recovery can sufficiently be made from adverse reactions through medical treatment, was higher in level for groups closer to vaccination without hesitancy; thus, it had a positive impact on vaccination. All factors showed statistical significance in the univariate analyses (Table 2), so the HBM-VH decision model was valid. The multivariate analysis chose a logistic regression rather than combined analyses, such as a structural equation model, considering the small sample size. As a result, the perception that the child could experience infections leading to severe conditions when not vaccinated, which was an item of perceived severity, was significant to accept vaccines (Table 3). To identify the reason for the group differences in the perception of this factor, a large-sample between-group comparison study or qualitative research on the refusal group is necessary.

**Figure 2.**
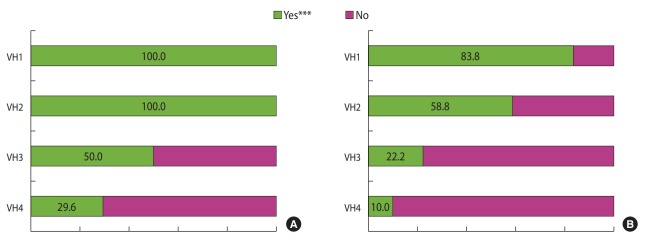
Levels of perception about the (A) necessity and (B) safety of child vaccinations. VH1, vaccination without hesitancy; VH2, vaccination on time with hesitancy; VH3, vaccination with a deliberate delay of one month or longer (one or more); VH4, vaccination refusal (one or more). ***p<0.001 from chi-square for trend test.

Delayed or refused Japanese encephalitis vaccine, DTaP, MMR, and IPV were slightly more common. The first dose of DTaP and IPV start two months after birth, there are a number of doses until the age of four to six. The first dose of Japanese encephalitis vaccine and MMR come after 12 months. In contrast, the number of refusals for BCG and hepatitis B, which are vaccinated immediately after birth, was relatively low. These orders are assumed to be given because personal experiences with adverse reactions affected future vaccination refusal. Similar items of HBM were significant as well. Additionally, for all three groups of vaccination with hesitancy, delay, and refusal, the concerns and experiences of adverse reactions were the greatest reason for hesitation at 65.0-75.8% (Table 4), trust in the safety decreased (Figure 2), and more than half of all four VH groups responded that the vaccination information was insufficient. These results showed the importance of advertisement and communication. Additionally, when considering the distrust in the vaccination safety management and government policies (Table 4), understanding of the vaccination policies and management systems along with information about adverse reactions is important for communication. Being provided with sufficient information and communicating with experts are the rights of the parents who decide vaccination and are important factors. There are various overseas studies on which approach is effective, and the results varied due to the differences in the social systems and cultures of each country [5,8,10]. Korean research is needed to identify suitable approachs.

As for reasons to hesitate and refuse vaccination, the concerns and experiences of adverse reactions, distrust of policies and safety management, and not recognizing the meaningfulness of the infection prevention were the main reasons (Table 4). It can be hypothesized that the hesitant groups do not sufficiently trust the healthcare system. Furthermore, persons who affected vaccination refusal were online anti-vaccination activists, personnel of civil society groups, and experts of Korean traditional or alternative medicine. In particular, the impact of these people appeared greater for the refusal group. The effect of alternative medicine was reported in overseas studies as well [21]. However, distinguishment of Korean traditional medicine and alternative medicine is a task for the future studies because Korean official system includes traditional medicine. Additionally, the impact of online anti-vaccination activists was identified, similar to the previous studies [3,6,22]. To appropriately respond to the VH issue, health authorities and professionals must recognize these social movements and the impact of related persons [2]. Meanwhile, it must also be considered that criticism, regulation, and punishment-centered approaches and seeing citizens as the passive subjects of education/advertisement are ineffective in democratic countries [22-24]. Combining all of these facets, healthcare professionals must sufficiently and appropriately communicate with people who affect VH and affected parents [11,24-26].

The factors that led to ultimately accepting vaccination were doctors’ explanation, disadvantages to the unvaccinated child, and text messages and mails from institutions. Although overseas studies showed similar results that doctors are the most decisive [11,12], the contributed proportion of doctors did not exceed 30% in the group that vaccinated with hesitancy in this study. It is necessary to research the impact of the Korean clinical environment, with its short doctor consultations. In previous studies, general physicians played important roles in countries with strong primary healthcare and delivery systems, whereas pediatricians played important roles in countries that directly accessed pediatricians as primary caregivers [3,27]. The status and future plans of Korea should be studied considering them. Meanwhile, the proportion that decides to vaccinate due to concerns about the disadvantages faced by unvaccinated children is also high. There are researchs that regulation can have positive or negative effects on the rate of vaccination [12,28,29]. In particular, there is a tendency for the strengthening of regulations to have a negative effect in countries with stronger sovereignty of the people and a more stable democratic system [9,10,30]. Korean research that considers the historical, social, and cultural backgrounds is necessary [31].

Generally, the aim of VH research is to examine how to get parents to accept vaccines [1,5,7,22]. This is because if the hesitant population is large enough, there can be a ripple effect on the infection spread [30,32]. However, because Korea estimates that they are very small in number, it is not easy to make a significant ripple effects [13,14]. Also, heavy-handed regulation can increase the conflicts and avoidance rather than resolution [3,12,22,33]. Moreover, considering the research results that they have low trust in the healthcare system, caution must be taken in instituting regulation-based policies [34]. A resolution in which social agreement is possible must be found through comprehensive trust-building communication with VH parents [2,35].

VH researchs to date has mainly focused on Western countries and Japan, and status investigations or research in Korea have been insufficient. This research has limited representativeness because it was not a national survey and was conducted on a small number of parents in specific groups. However, this study can be a reference for large-scale or qualitative studies in the future, because this was conducted in alternative education facilities, in which VH parents are possibly concentrated. Particularly, the limitations in the representativeness and sample size was complmented by the HBM that examined how the positive and negative reasons for vaccination link to whether one vaccinates. The result found that VH factors are making impacts, similar to previous studies conducted in foreign countries. If status investigations show that there are hesitant peoples of considerable size with the possibility of expansion, like in other developed countries, this study can be an important reference for future studies and policy making. Meanwhile, Korea has characteristics such as official system for traditional medicine, an incomplete primary healthcare system and short consultations, and disadvantages about unvaccinated children. Future researchs are particularly important focusing on the Korea-specific situations.

## Figures and Tables

**Table 1.** The general characteristics of the parents by their perceptions about vaccinating children

Category	Outcome	No. of people	VH1	VH2	VH3	VH4	p-value^1^
Sex	Female	119	37 (88.1)	19 (95.0)	32 (100)	31 (91.2)	0.398
	Male	9	5 (11.9)	1 (5.0)	0 (0.0)	3 (8.8)	
	No response^2^	1	1	0	0	0	
Age (yr)	Mean±SD	38.1±3.7	37.5±3.6	38.9±4.0	38.5±4.2	37.8±3.2	0.460^3^
	Minimum-Maximum	28-46	30-46	29-44	28-46	32-45	
Marital status	Married	125	41 (95.3)	19 (95.0)	31 (100)	34 (100)	0.158
	Divorced or widowed	3	2 (4.7)	1 (5.0)	0 (0.0)	0 (0.0)	
	No response^2^	1	0	0	1	0	
Education level	Graduated high school	3	1 (2.3)	1 (5.0)	1 (3.1)	0 (0.0)	0.532
	Graduated college or university	95	31 (72.1)	11 (55.0)	26 (81.3)	27 (79.4)	
	Graduated graduate school	31	11 (25.6)	8 (40.0)	5 (15.6)	7 (20.6)	
Subjective income level	Upper	3	3 (7.1)	0 (0.0)	0 (0.0)	0 (0.0)	0.006
	Middle	109	36 (85.7)	19 (95.0)	30 (93.8)	24 (70.6)	
	Lower	14	3 (7.1)	1 (5.0)	2 (6.3)	8 (23.5)	
	No response^2^	3	1	0	0	2	
No. of children	1	47	17 (39.5)	10 (50.0)	8 (25.0)	12 (35.3)	0.355
	2	69	22 (51.2)	9 (45.0)	20 (62.5)	18 (52.9)	
	3	13	4 (9.3)	1 (5.0)	4 (12.5)	4 (11.8)	
Total		129 (100)	43 (33.3)	20 (15.5)	32 (24.8)	34 (26.4)	-

Values are presented as number (%). VH1, vaccination without hesitancy; VH2, vaccination on time with hesitancy; VH3, vaccination with a deliberate delay of one month or longer (one or more); VH4, vaccination refusal (one or more); SD, standard deviation.

1 Chi-square for trend test.

2 No response are not included in the percentage.

3 Age– analysis of variance.

**Table 2.** Levels of factors of the health belief model by vaccine hesitancy

Questions	Agreed/respondents	VH1	VH2	VH3	VH4	p-value^1^
Perceived susceptibility	79/124 (63.7)	39 (90.7)	16 (80.0)	14 (46.7)	10 (32.3)	<0.001
If my child does not receive the required vaccinations, she/he could be affected by infectious diseases						
I don’t know^2^	5	0	0	2	3	
Perceived severity	62/121 (51.2)	36 (83.7)	8 (50.0)	11 (36.7)	7 (21.9)	<0.001
If my child does not receive the required vaccinations, she/he could develop a severe condition (example: inpatient treatment at a medical facility) due to infectious diseases						
I don’t know^2^	8	0	4	2	2	
Even if my child does not receive the required vaccinations and is affected by infectious diseases, she/he can sufficiently recover naturally without medical treatment	36/113 (31.9)	6 (14.3)	3 (20.0)	12 (42.9)	15 (53.6)	<0.001
I don’t know^2^	16	1	5	4	6	
Perceived benefits	72/117 (61.5)	41 (95.3)	15 (78.9)	12 (44.4)	4 (14.3)	<0.001
If my child receives the required vaccinations, infectious diseases can be effectively prevented						
I don’t know^2^	12	0	1	5	6	
Perceived barriers	104/120 (86.7)	30 (76.9)	16 (84.2)	28 (93.3)	30 (93.8)	0.024
If my child receives the required vaccinations, adverse reactions might occur						
I don’t know^2^	9	4	1	2	2	
I have not received trustable and sufficient information about the required vaccinations for my child	88/126 (69.8)	23 (56.1)	12 (63.2)	27 (84.4)	26 (76.5)	0.019
I don’t know^2^	3	2	1	0	0	
Cues to action	28/129 (21.7)	2 (4.7)	4 (20.0)	11 (34.4)	11 (32.4)	0.001
Have your child actually had an experience of adverse reactions after receiving the required vaccination?						
I don’t know^2^	0	0	0	0	0	
Have you directly or indirectly heard of cases in which the child of some- one close to you has delayed or refused the required vaccination?	101/129 (78.3)	25 (58.1)	17 (85.0)	28 (87.5)	31 (91.2)	<0.001
I don’t know^2^	0	0	0	0	0	
Have you directly or indirectly heard of cases in which the child of some- one close to you has experienced adverse reactions after receiving the required vaccination?	52/129 (40.3)	7 (16.3)	10 (50.0)	18 (56.3)	17 (50.0)	0.001
I don’t know^2^	0	0	0	0	0	
Self-efficacy	54/102 (52.9)	27 (81.8)	10 (62.5)	9 (34.6)	8 (29.6)	<0.001
Even if my child experiences adverse reactions due to required vac- cinations, she/he can recover through sufficient medical treatment						
I don’t know^2^	27	10	4	6	7	
Total	129 (100)	43 (100)	20 (100)	32 (100)	34 (100)	

Values are presented as number (%). VH1, vaccination without hesitancy, in time; VH2, vaccination with hesitancy, in time; VH3, vaccination with hesitancy, intentionally, over 1 month delayed; VH4, vaccination refusal, intentionally, over one vaccine.

1 Chi-square for trend test.

2 Respondents of ‘I don’t know’ are not included in the percentage.

**Table 3.** Results of a logistic regression analysis on the factors of vaccine hesitancy

Questions		Unadjusted	Adjusted^1^
Perceived severity			
If my child does not receive the required vaccinations, she/he could develop a severe condition (example: inpatient treatment at a medical facility) due to infectious diseases.	Disagree	1.0 (reference)	1.0 (reference)
	Agree	7.2 (3.2, 16.0)	6.5 (1.1, 38.9)
Even if my child does not receive the required vaccinations and is affected by infectious diseases, she/he can sufficiently recover naturally without medical treatment.	Disagree	1.0 (reference)	1.0 (reference)
	Agree	5.0 (2.1, 12.0)	1.3 (0.2, 7.4)
Perceived benefits			
If my child receives the required vaccinations, infectious diseases can be effectively prevented.	Agree	1.0 (reference)	1.0 (reference)
	Disagree	22.8 (8.2, 63.3)	5.0 (0.9, 28.6)
Perceived barriers			
If my child receives the required vaccinations, adverse reactions might occur.	Disagree	1.0 (reference)	1.0 (reference)
	Agree	3.8 (1.1, 12.5)	2.7 (0.4, 18.1)
I have not received trustable and sufficient information about the required vaccinations for my child.	Disagree	1.0 (reference)	1.0 (reference)
	Agree	2.9 (1.3, 6.4)	1.0 (0.2, 4.8)
Cue to action			
Have your child actually had an experience of adverse reactions after receiving the required vaccination?	No	1.0 (reference)	1.0 (reference)
	Yes	4.8 (1.8, 12.7)	2.0 (0.2, 19.3)
Have you directly or indirectly heard of cases in which the child of someone close to you has delayed or refused the required vaccination?	No	1.0 (reference)	1.0 (reference)
	Yes	4.2 (1.6, 10.8)	4.1 (0.6, 28.0)
Have you directly or indirectly heard of cases in which the child of someone close to you has experienced adverse reactions after receiving the required vaccination?	No	1.0 (reference)	1.0 (reference)
	Yes	3.1 (1.5, 6.4)	3.4 (0.6, 20.0)
Self-efficacy			
Even if my child experiences adverse reactions due to required vaccinations, she/he can recover through sufficient medical treatment.	Agree	1.0 (reference)	1.0 (reference)
	Disagree	6.5 (2.7, 15.6)	1.3 (0.2, 7.4)

Values are presented as odds ratio (95% confidence interval).

1 Adjusted: Logistic regression model (entered), explanatory power 67.1%, goodness-of-fit test p=0.509.

**Table 4.** Reasons for vaccine hesitancy

Reasons for vaccine hesitancy	Classification by vaccine hesitancy (responded “yes”)^1^		
	VH2	VH3	VH4
Because I do not think that vaccination meaningfully prevents infectious diseases	4 (20.0)	12 (38.7)	20 (58.8)
Because of concerns about the adverse reactions caused by vaccinations or because of prior experiences with small or large adverse reactions	13 (65.0)	22 (68.8)	25 (75.8)
Because I cannot trust the vaccination policies of the government	5 (25.0)	17 (53.1)	18 (54.5)
Because I cannot trust the vaccination safety management of pharmaceutical companies and medical institutions	10 (50.0)	15 (46.9)	22 (68.8)
Because of religious beliefs	0 (0.0)	0 (0.0)	0 (0.0)
Because I trust Korean traditional medicine or alternative medicine more than modern medical science, or because of naturalistic beliefs	3 (15.0)	7 (21.9)	7 (21.2)
Total	20 (100)	32 (100)	43 (100)

Values are presented as number (%). VH2, vaccination with hesitancy, in time; VH3, vaccination with hesitancy, intentionally, over 1 month delayed; VH4, vaccination refusal, intentionally, over one vaccine.

1 Multiple responses possible per item.

**Table 5.** Person(s) who had an impact on vaccine hesitancy

Person(s) who had an impact on the vaccine hesitancy	Classification by vaccine hesitancy (responded with “yes”)^1^		
	VH1	VH2	VH3
Family (spouse, parents, male siblings, female siblings, cousins, etc.)	1 (5.0)	11 (34.4)	6 (17.6)
Acquaintances (friends, colleagues, neighbors, etc.)	8 (40.0)	11 (34.4)	18 (52.9)
Leaders or activists of a joined organization (social groups, civil society groups, etc.)	2 (10.0)	6 (18.8)	13 (39.4)
Leaders or activists of a joined organization (religious group)	0 (0.0)	0 (0.0)	0 (0.0)
Anti-vaccination activists whom I found through web, blogs, social network services, and broadcasts	11 (55.0)	12 (37.5)	18 (54.5)
Experts of Korean traditional medicine or alternative medicine	1 (5.0)	8 (25.0)	10 (30.3)
Decision made by myself	10 (50.0)	25 (78.1)	25 (75.8)
Total	20 (100)	32 (100)	43 (100)

VH1, Vaccination without hesitancy, in time; VH2, vaccination with hesitancy, in time; VH3, vaccination with hesitancy, intentionally, over 1 month delayed.

1 Multiple responses possible per item.

## SUPPLEMENTARY MATERIALS

Korean version is available at http://www.e-epih.org/.
