# Supplementary material for: Why do some Korean parents hesitate to vaccinate their children?
Source: Epidemiol Health. 2019 Jul 9;41:e2019031. doi: 10.4178/epih.e2019031 (PMC6661469; doi:10.4178/epih.e2019031)
Supplement: Supplementary file 1 [file epih-41-e2019031-supplementary1.pdf]

# <조사 설문지>

다음의 질문들을 주의 깊게 읽으신 후 답변하여 주시면 감사하겠습니다.

| 기본 질문 | | |
|---|---|---|
| **본인<br>기본 정보** | 1. 성별<br>2. 나이<br>3. 결혼 여부<br>4. 거주지<br>5. 교육 수준<br>6. 소득 수준<br>(본인 주관적) | 1. ① 여성 ② 남성<br>2. 만 ____세<br>3. ① 비혼 ② 기혼 ③ 이혼, 사별<br>4. 광역시도: ________, 시군구: _________<br>5. ① 중졸 이하 ② 고졸 ③ 대졸 ④ 대학원졸<br>6. ① 상 ② 중 ③ 하 |
| **자녀<br>기본 정보** | 1. 자녀 수<br>2. 자녀 동거 여부<br>3. 자녀 질병 | 1. ____명 (그 중 2010년생 이후 ____명)<br>2. ① 동거 ② 비동거<br>3. ① 예방접종이 불가능한 의학적 질병 있(었)음<br>② 그러한 질병 없음 |

| 공통 질문 | |
|---|---|
| 1. 당신 자녀(들)의 필수예방접종을 맞추는 결정을 할 때 **접종을 망설이거나, 의학적으로 어쩔 수 없이 못 맞추는 상황(즉, 건강 상태나 질병 치료 등으로 불가피하게 맞추지 못하는 상황)이 아닌데도 의도적으로 미루거나, 혹은 맞추지 않은 경험**이 있습니까?<br><br>□ ① **망설임 없이** 필수예방접종을 모두 제 때에 맞추었다.<br>(늦출 의도가 없었는데 바쁘거나 깜박해서 지연하거나 못 맞춘 경우 → ①에 포함)<br>□ ② **(망설임 접종)** 망설인 적은 있지만 모두 제 때에 맞추었다.<br>□ ③ **(지연 접종)** 망설인 적이 있으며, 1개 이상을 1개월 이상 지연하여 맞추었다.<br>(여러 번 맞추는 경우 한 번이라도 해당되면 체크해주세요)<br>□ ④ **(미접종)** 1개 이상의 필수예방접종을 맞추지 않았다.<br>(여러 번 맞추는 경우 한 번이라도 해당되면 체크해주세요) | |
| 2. 내 아이가 필수 예방접종을 맞지 않으면, **전염병에 걸릴 수 있다.** | ① 매우 동의함<br>② 동의함<br>③ 동의하지 않음<br>④ 매우 동의하지 않음<br>⑤ 모르겠음 |

| | |
|---|---|
| 3. 내 아이가 필수 예방접종을 맞지 않으면, **전염병에 걸려 심각한 상태**(예: 의료기관 입원 치료)**에 이를 수 있다.** | ① 매우 동의함<br>② 동의함<br>③ 동의하지 않음<br>④ 매우 동의하지 않음<br>⑤ 모르겠음 |
| 4. 내 아이가 필수 예방접종을 맞으면, **효과적으로 전염병을 예방할 수 있다.** | ① 매우 동의함<br>② 동의함<br>③ 동의하지 않음<br>④ 매우 동의하지 않음<br>⑤ 모르겠음 |
| 5-1. 내 아이가 필수 예방접종을 맞으면, **백신 부작용이 발생할 수 있다.** | ① 매우 동의함<br>② 동의함<br>③ 동의하지 않음<br>④ 매우 동의하지 않음<br>⑤ 모르겠음 |
| 5-2. 내 아이의 필수 **예방접종에 대한 신뢰**할만한 **충분한 정보를 제공 받은 적이 없다.** | ① 매우 동의함<br>② 동의함<br>③ 동의하지 않음<br>④ 매우 동의하지 않음<br>⑤ 모르겠음 |
| 6. 실제로 당신의 자녀가 어린이 필수 예방접종 이후 **부작용을 겪은 경험**이 있습니까? | ① 예 ② 아니오 |
| 7-1. 나와 가까운 사람의 자녀가 어린이 필수 예방접종을 **지연하거나 맞추지 않은 경우**를 직·간접으로 들은 적이 있습니까? | ① 예 ② 아니오 |
| 7-2. 나와 가까운 사람의 자녀가 어린이 필수 예방접종 이후 **부작용**을 겪은 경우를 직·간접으로 들은 적이 있습니까? | ① 예 ② 아니오 |
| 8. 내 아이가 필수 **예방접종으로 부작용**이 발생하여도, **충분한 의학적 조치를 받아서 완쾌할 수 있다.** | ① 매우 동의함<br>② 동의함<br>③ 동의하지 않음<br>④ 매우 동의하지 않음<br>⑤ 모르겠음 |
| 9. 내 아이가 필수 **예방접종을 맞지 않고 전염병**에 걸리더라도, 병원 치료 없이 **자연적으로 충분히 회복할 수 있다.** | ① 매우 동의함<br>② 동의함<br>③ 동의하지 않음<br>④ 매우 동의하지 않음<br>⑤ 모르겠음 |

| | |
|---|---|
| 10. 어린이 필수 예방접종의 **<u>필요성(=자녀를 전염병으로부터 보호할 수 있음</u>**)을 **<u>얼마나 신뢰</u>**하십니까?<br>(= 필수 예방접종을 맞으면 전염병에 안 걸리기 때문에 반드시 필요하다.) | ① 매우 필요함<br>② 필요함<br>③ 필요하지 않음<br>④ 매우 필요하지 않음<br>⑤ 모르겠음 |
| 11. 어린이 필수 예방접종의 **<u>안전성(=치명적 백신 부작용에 대한 우려 없음</u>**)을 **<u>얼마나 신뢰</u>**하십니까?<br>(= 필수 예방접종을 맞은 경우, 부작용이 일어날 가능성이 거의 없어서 안전하다.) | ① 매우 안전함<br>② 안전함<br>③ 안전하지 않음<br>④ 매우 안전하지 않음<br>⑤ 모르겠음 |

① 망설임 없이 필수예방접종을 모두 제 때에 맞추었다. ⇒ <u>1형 설문지</u>

(늦출 의도가 없었는데 바쁘거나 깜박해서 지연하거나 못 맞춘 경우 → ①에 포함)

② 망설인 적은 있지만 모두 제 때에(1개월 이내 포함) 맞추었다. ⇒ <u>2형 설문지</u>

③ 망설인 적이 있으며, 1개 이상을 1개월 이상 지연하여 맞추었다. ⇒ <u>3형 설문지</u>

④ 1개 이상의 필수예방접종을 맞추지 않았다. ⇒ <u>4형 설문지</u>

**① 망설임 없이 필수예방접종을 모두 제 때에 맞추었다. ⇒ 1형 설문지**

② (망설임 접종) 망설인 적은 있지만 모두 제 때에(1개월 이내 포함) 맞추었다. ⇒ 2형 설문지
③ (지연 접종) 망설인 적이 있으며, 1개 이상을 1개월 이상 지연하여 맞추었다. ⇒ 3형 설문지
④ (미접종) 1개 이상의 필수예방접종을 맞추지 않았다. ⇒ 4형 설문지

| **1형 설문지 - 망설임 없이 모두 제 때에 접종한 경우**<br>**(각 세부 질문의 예/아니오에 각각 답변해주세요)** | |
|---|---|
| 1. 귀하가 자녀의 **예방접종을 일정대로 맞추게 되는데 가장 영향을 준 사람이나 이유**로 다음에 해당하는 것이 있습니까? | |
| 1-1. 자녀를 진료한 의사(소아과, 가정의학과, 내과 등)의 설명 때문 | 예( ), 아니오( ) |
| 1-2. 자녀를 진료한 한의사의 설명 때문 | 예( ), 아니오( ) |
| 1-3. 의료기관에서 만난 간호사의 설명 때문 | 예( ), 아니오( ) |
| 1-4. 그 외 보건의료 종사자들의 설명 때문 | 예( ), 아니오( ) |
| 1-5. 정부 정책 비협조 시 자녀가 받을 불이익을 우려했기 때문<br>(예: 일부 접종은 미접종 자녀 초등학교 입학 시 문제가 될 수 있음) | 예( ), 아니오( ) |
| 1-6. 인터넷, 책, 방송 등을 통한 예방접종 홍보 때문 | 예( ), 아니오( ) |
| 1-7. 가족이나 지인의 설명 때문 | 예( ), 아니오( ) |
| 1-8. 부모 스스로의 판단 때문 | 예( ), 아니오( ) |
| 1-9. 병의원이나 보건소 등 기관에서 보내는 안내 문자, 우편물 때문 | 예( ), 아니오( ) |
| 1-10. 기타: ________________________________ | 예( ), 아니오( ) |

1형 설문지에 해당하는 분들은 여기까지 답변하시면 됩니다.

## <완료 감사 및 추가 참여자 모집 안내>

설문을 완료하여 주셔서 대단히 감사합니다.
문의사항이 있으시면 언제든지 연구자 장규진(직업환경의학 전문의, 아주의대 박사 과정, doctor25067@gmail.com)에게 연락해주세요.

**② (망설임 접종) 망설인 적은 있지만 모두 제 때에(1개월 이내 포함) 맞추었다.**
**⇒ 2형 설문지**

① 망설임 없이 필수예방접종을 모두 제 때에 맞추었다. ⇒ 1형 설문지
③ (지연 접종) 망설인 적이 있으며, 1개 이상을 1개월 이상 지연하여 맞추었다. ⇒ 3형 설문지
④ (미접종) 1개 이상의 필수예방접종을 맞추지 않았다. ⇒ 4형 설문지

| **2형 설문지 - 망설인 적은 있지만 모두 제 때에 접종한 경우**<br>(각 세부 질문의 예/아니오에 각각 답변해주세요) | |
|---|---|
| 1. 만약 **특정 예방접종을 망설인 경우**가 있다면 **어떤 접종**입니까? (해당되는 것 모두)<br>☐ ① BCG(결핵, 피내용이나 경피용) ☐ ② B형간염<br>☐ ③ DTaP(디프테리아, 파상풍, 백일해) ☐ ④ IPV(소아마비)<br>☐ ⑤ MMR(홍역, 유행성이하선염, 풍진) ☐ ⑥ 수두<br>☐ ⑦ 일본뇌염 ☐ ⑧ 어떤 것인지는 모르지만 있었음 | |
| 2. 자녀의 예방접종을 **망설였던 이유** 중 다음에 해당하는 것이 있습니까? | |
| 2-1. 예방접종이 전염병 예방에 의미 있다고 생각하지 않기 때문 | 예( ), 아니오( ) |
| 2-2. 예방접종 부작용의 우려나 과거 크고 작은 부작용 경험 때문 | 예( ), 아니오( ) |
| 2-3. 정부의 예방접종 정책을 믿을 수 없기 때문 | 예( ), 아니오( ) |
| 2-4. 제약회사·의료기관의 예방접종 안전관리를 믿을 수 없기 때문 | 예( ), 아니오( ) |
| 2-5. 종교적 신념 때문 | 예( ), 아니오( ) |
| 2-6. 현대의학보다 한의학이나 대체의학 등을 더 신뢰하기 때문,<br>혹은 자연주의 신념 때문 | 예( ), 아니오( ) |
| 2-7. 기타: ________________________________ | 예( ), 아니오( ) |
| 3. 자녀의 예방접종을 **망설이는데 영향을 준 사람**으로 다음에 해당하는 경우가 있습니까? | |
| 3-1. 가족(배우자, 부모, 형제, 자매, 친척 등) | 예( ), 아니오( ) |
| 3-2. 지인(친구, 동료, 이웃 등) | 예( ), 아니오( ) |
| 3-3. 소속 단체(사회 모임, 시민단체 등) 지도자 혹은 활동가 | 예( ), 아니오( ) |
| 3-4. 소속 단체(종교 모임) 지도자 혹은 활동가 | 예( ), 아니오( ) |
| 3-5. 인터넷/블로그/SNS/책/방송 등을 통해 접한 백신 반대 활동가 | 예( ), 아니오( ) |

| | |
|---|---|
| 3-6. 한의학이나 대체의학 전문가 | 예( ), 아니오( ) |
| 3-7. 스스로 결정 | 예( ), 아니오( ) |
| 3-8. 기타: ________________________________ | 예( ), 아니오( ) |
| 4. **<u>망설이기는 했지만 결국 예방접종을 일정대로 접종하도록 결정한 이유</u>**로 다음에 해당하는 것이 있습니까? (미접종 시 전염병 위험성 등을 이유로 설득한 사람 혹은 계기) | |
| 4-1. 자녀를 진료한 의사(소아과, 가정의학과, 내과 등)의 설득 때문 | 예( ), 아니오( ) |
| 4-2. 자녀를 진료한 한의사의 설득 때문 | 예( ), 아니오( ) |
| 4-3. 의료기관에서 만난 간호사의 설득 때문 | 예( ), 아니오( ) |
| 4-4. 그 외 보건의료 종사자들의 설득 때문 | 예( ), 아니오( ) |
| 4-5. 정부 정책 비협조 시 자녀가 받을 불이익을 우려했기 때문 (예: 일부 접종은 미접종 자녀 초등학교 입학 시 문제가 될 수 있음) | 예( ), 아니오( ) |
| 4-6. 인터넷, 책, 방송 등을 통한 예방접종 홍보 때문 | 예( ), 아니오( ) |
| 4-7. 가족이나 지인의 설득 때문 | 예( ), 아니오( ) |
| 4-8. 부모 스스로의 판단 때문 | 예( ), 아니오( ) |
| 4-9. 병의원이나 보건소 등 기관에서 보내는 안내 문자, 우편물 때문 | 예( ), 아니오( ) |
| 4-10. 기타: ________________________________ | 예( ), 아니오( ) |

2형 설문지에 해당하는 분들은 여기까지 답변하시면 됩니다.

## <완료 감사 및 추가 참여자 모집 안내>

설문을 완료하여 주셔서 대단히 감사합니다.
문의사항이 있으시면 언제든지 연구자 장규진(직업환경의학 전문의, 아주의대 박사 과정, doctor25067@gmail.com)에게 연락해주세요.

**③ (지연 접종) 망설인 적이 있으며, 1개 이상을 1개월 이상 지연하여 맞추었다.**
**⇒ 3형 설문지**

① 망설임 없이 필수예방접종을 모두 제 때에 맞추었다. ⇒ 1형 설문지
② (망설임 접종) 망설인 적은 있지만 모두 제 때에(1개월 이내 포함) 맞추었다. ⇒ 2형 설문지
④ (미접종) 1개 이상의 필수예방접종을 맞추지 않았다. ⇒ 4형 설문지

| | |
|---|---|
| **3형 설문지 - 망설여서 1개월 이상 지연한 것도 있으나 결국 접종을 한 경우 (여러 번 맞을 경우 한 번이라도 포함되면 해당)**<br>(각 세부 질문의 예/아니오에 각각 답변해주세요) | |
| 1. 만약 **특정 예방접종을 지연한 경우**가 있다면 **어떤 접종**입니까? (해당되는 것 모두)<br>□ ① BCG(결핵, 피내용이나 경피용) □ ② B형간염<br>□ ③ DTaP(디프테리아, 파상풍, 백일해) □ ④ IPV(소아마비)<br>□ ⑤ MMR(홍역, 유행성이하선염, 풍진) □ ⑥ 수두<br>□ ⑦ 일본뇌염 □ ⑧ 어떤 것인지는 모르지만 있었음 | |
| 2. 자녀의 예방접종을 **지연하였던 이유** 중 다음에 해당하는 것이 있습니까? | |
| 2-1. 예방접종이 전염병 예방에 의미 있다고 생각하지 않기 때문 | 예( ), 아니오( ) |
| 2-2. 예방접종 부작용의 우려나 과거 크고 작은 부작용 경험 때문 | 예( ), 아니오( ) |
| 2-3. 정부의 예방접종 정책을 믿을 수 없기 때문 | 예( ), 아니오( ) |
| 2-4. 제약회사·의료기관의 예방접종 안전관리를 믿을 수 없기 때문 | 예( ), 아니오( ) |
| 2-5. 종교적 신념 때문 | 예( ), 아니오( ) |
| 2-6. 현대의학보다 한의학이나 대체의학 등을 더 신뢰하기 때문,<br>혹은 자연주의 신념 때문 | 예( ), 아니오( ) |
| 2-7. 기타: ______________________________ | 예( ), 아니오( ) |
| 3. 자녀의 예방접종을 **지연하는데 영향을 준 사람**으로 다음에 해당하는 경우가 있습니까? | |
| 3-1. 가족(배우자, 부모, 형제, 자매, 친척 등) | 예( ), 아니오( ) |
| 3-2. 지인(친구, 동료, 이웃 등) | 예( ), 아니오( ) |
| 3-3. 소속 단체(사회 모임, 시민단체 등) 지도자 혹은 활동가 | 예( ), 아니오( ) |
| 3-4. 소속 단체(종교 모임) 지도자 혹은 활동가 | 예( ), 아니오( ) |

| | |
|---|---|
| 3-5. 인터넷/블로그/SNS/책/방송 등을 통해 접한 백신 반대 활동가 | 예( ), 아니오( ) |
| 3-6. 한의학이나 대체의학 전문가 | 예( ), 아니오( ) |
| 3-7. 스스로 결정 | 예( ), 아니오( ) |
| 3-8. 기타: ______________________________ | 예( ), 아니오( ) |
| 4. **지연하기는 했지만 결국 예방접종을 늦게라도 접종하도록 결정한 이유**로 다음에 해당하는 것이 있습니까? (미접종 시 전염병 위험성 등을 이유로 설득한 사람 혹은 계기) | |
| 4-1. 자녀를 진료한 의사(소아과, 가정의학과, 내과 등)의 설득 때문 | 예( ), 아니오( ) |
| 4-2. 자녀를 진료한 한의사의 설득 때문 | 예( ), 아니오( ) |
| 4-3. 의료기관에서 만난 간호사의 설득 때문 | 예( ), 아니오( ) |
| 4-4. 그 외 보건의료 종사자들의 설득 때문 | 예( ), 아니오( ) |
| 4-5. 정부 정책 비협조 시 자녀가 받을 불이익을 우려했기 때문 (예: 일부 접종은 미접종 자녀 초등학교 입학 시 문제가 될 수 있음) | 예( ), 아니오( ) |
| 4-6. 인터넷, 책, 방송 등을 통한 예방접종 홍보 때문 | 예( ), 아니오( ) |
| 4-7. 가족이나 지인의 설득 때문 | 예( ), 아니오( ) |
| 4-8. 부모 스스로의 판단 때문 | 예( ), 아니오( ) |
| 4-9. 병의원이나 보건소 등 기관에서 보내는 안내 문자, 우편물 때문 | 예( ), 아니오( ) |
| 4-10. 기타: ______________________________ | 예( ), 아니오( ) |

3형 설문지에 해당하는 분들은 여기까지 답변하시면 됩니다.

## <완료 감사 및 추가 참여자 모집 안내>

설문을 완료하여 주셔서 대단히 감사합니다.
문의사항이 있으시면 언제든지 연구자 장규진(직업환경의학 전문의, 아주의대 박사 과정, doctor25067@gmail.com)에게 연락해주세요.

**④ (미접종) 1개 이상의 필수예방접종을 맞추지 않았다. ⇒ 4형 설문지**

① 망설임 없이 필수예방접종을 모두 제 때에 맞추었다. ⇒ 1형 설문지
② (망설임 접종) 망설인 적은 있지만 모두 제 때에(1개월 이내 포함) 맞추었다. ⇒ 2형 설문지
③ (지연 접종) 망설인 적이 있으며, 1개 이상을 1개월 이상 지연하여 맞추었다. ⇒ 3형 설문지

**4형 설문지 - 1개 이상 필수예방접종을 결국 맞추지 않은 경우**
**(여러 번 맞을 경우 한 번이라도 포함되면 해당)**
**(각 세부 질문의 예/아니오에 각각 답변해주세요)**

1. 만약 **특정 예방접종을 맞추지 않은 경우**가 있다면 **어떤 접종**입니까?
(해당되는 것 모두)

☐ ① BCG(결핵, 피내용이나 경피용)
☐ ② B형간염
☐ ③ DTaP(디프테리아, 파상풍, 백일해)
☐ ④ IPV(소아마비)
☐ ⑤ MMR(홍역, 유행성이하선염, 풍진)
☐ ⑥ 수두
☐ ⑦ 일본뇌염
☐ ⑧ 어떤 것인지는 모르지만 있었음

2. 자녀의 예방접종을 **맞추지 않은 이유** 중 다음에 해당하는 것이 있습니까?

| | |
|---|---|
| 2-1. 예방접종이 전염병 예방에 의미 있다고 생각하지 않기 때문 | 예( ), 아니오( ) |
| 2-2. 예방접종 부작용의 우려나 과거 크고 작은 부작용 경험 때문 | 예( ), 아니오( ) |
| 2-3. 정부의 예방접종 정책을 믿을 수 없기 때문 | 예( ), 아니오( ) |
| 2-4. 제약회사·의료기관의 예방접종 안전관리를 믿을 수 없기 때문 | 예( ), 아니오( ) |
| 2-5. 종교적 신념 때문 | 예( ), 아니오( ) |
| 2-6. 현대의학보다 한의학이나 대체의학 등을 더 신뢰하기 때문, 혹은 자연주의 신념 때문 | 예( ), 아니오( ) |
| 2-7. 기타: ______________________ | 예( ), 아니오( ) |

3. 자녀의 예방접종을 **맞추지 않는데 영향을 준 사람**으로 다음에 해당하는 경우가 있습니까?

| | |
|---|---|
| 3-1. 가족(배우자, 부모, 형제, 자매, 친척 등) | 예( ), 아니오( ) |
| 3-2. 지인(친구, 동료, 이웃 등) | 예( ), 아니오( ) |
| 3-3. 소속 단체(사회 모임, 시민단체 등) 지도자 혹은 활동가 | 예( ), 아니오( ) |
| 3-4. 소속 단체(종교 모임) 지도자 혹은 활동가 | 예( ), 아니오( ) |

| | |
|---|---|
| 3-5. 인터넷/블로그/SNS/책/방송 등을 통해 접한 백신 반대 활동가 | 예( ), 아니오( ) |
| 3-6. 한의학이나 대체의학 전문가 | 예( ), 아니오( ) |
| 3-7. 스스로 결정 | 예( ), 아니오( ) |
| 3-8. 기타: ______________________________ | 예( ), 아니오( ) |

4형 설문지에 해당하는 분들은 여기까지 답변하시면 됩니다.

## <완료 감사 및 추가 참여자 모집 안내>

설문을 완료하여 주셔서 대단히 감사합니다.
문의사항이 있으시면 언제든지 연구자 장규진(직업환경의학 전문의, 아주의대 박사 과정, doctor25067@gmail.com)에게 연락해주세요.

# <참조 - 예방접종 일정표>

2010년대 이후 태어난 한국 어린이들의 필수 예방접종은 다음과 같습니다.
(2017년 개정으로 좀 더 늘어났으나, 아래는 2010년 기준입니다.)

| 종 류 | 1개월 이내 | 1개월 | 2개월 | 4개월 | 6개월 | 12-15 개월 | 만4-6 세 |
|---|---|---|---|---|---|---|---|
| 결핵(BCG) | 1회 | | | | | | |
| B형간염 | 1차 | 2차 | | | 3차 | | |
| DTaP<br>(디프테리아, 파상풍, 백일해) | | | 1차 | 2차 | 3차 | 4차 | 5차 |
| IPV(소아마비) | | | 1차 | 2차 | 3차 | | 4차 |
| MMR<br>(홍역, 유행성 이하선염, 풍진) | | | | | | 1차 | 2차 |
| 수두 | | | | | | 1회 | |
| 일본뇌염 | 사백신: 5회 - 생후 12-36개월 (1, 2, 3차), 만6세, 만12세<br>혹은<br>약독화 생백신: 2회 - 생후 12-36개월 (1차), 만 6세 (2차) | | | | | | |
