# Supplementary material for: Why do some Korean parents hesitate to vaccinate their children?
Source: Epidemiol Health. 2019 Jul 9;41:e2019031. doi: 10.4178/epih.e2019031 (PMC6661469; doi:10.4178/epih.e2019031)
Supplement: Supplementary file 2 [file epih-41-e2019031-supplementary2.pdf]

Open Access

Volume: 41, Article ID: e2019031
https://doi.org/10.4178/epih.e2019031

epiH Epidemiology and Health

ORIGINAL ARTICLE

소아 예방접종 망설임의 이유

장규진, 이순영

아주대학교 의과대학 예방의학교실

Why do some Korean parents hesitate to vaccinate their children?

Kyujin Chang, Soon Young Lee

Department of Preventive Medicine and Public Health, Ajou University School of Medicine, Suwon, Korea

OBJECTIVES: Vaccinations for infectious diseases are opposed despite their achievement, and this opposition has recently been revealed in Korea. However, research in Korea has not been vigorous. The authors studied why some Korean parents hesitate to vaccinate their children by applying the health belief model.

METHODS: Parents who hesitate to vaccinate and parents who do not were surveyed in alternative education preschools and elementary schools. They were classified into four types of hesitancy and statistically compared.

RESULTS: Among the 129 subjects, 43 vaccinated without hesitancy, 20 vaccinated on time with hesitancy, 32 vaccinated with a deliberate delay of one month or longer, and 34 did not vaccinate. Vaccination increased with an increase in the awareness that severe outcomes can occur when unvaccinated. Concerns about adverse reactions from vaccinations or direct/indirect experiences affected refusal. Furthermore, perceptions of the lack of meaningfulness of vaccinations, distrust of policy and safety management, influence of leaders or activists in joined organizations, and experts of Korean traditional or alternative medicine affected refusal. Explanations by doctors, text messages and mails from institutions, and concerns about disadvantages caused by not complying with government policies increased vaccination.

CONCLUSIONS: The reasons for vaccine hesitancy and acceptance were similar to the results of international research. Health authorities and professionals should communicate sufficiently and appropriately with hesitant parents and find ways to rationally resolve social conflicts. However, this sample was small and there is little Korean research, so more in-depth and diverse researches are needed.

KEY WORDS: Vaccine hesitancy, Vaccination refusal, Health belief model, Korea

서 론

감염병 예방접종은 인류가 현대 과학을 통해 획득한 성과로서, 어린이들의 생명과 건강을 지키는 필수 도구로 인식된다[1]. 하지만 18세기 최초의 두창 예방접종 때부터 현대에 이르기까지 여러 국가들에서 예방접종을 수용하지 않는 흐름이 지속되어 왔다[2, 3]. 특히 미국, 유럽, 호주, 일본 등 주요 선진국들은 충분히 예방접종의 사회경제적 장벽을 제거했음에도 의도적 예방접종 반대 집단이 지금도 상당한 규모로 존재한다[1, 4-6]. 또한 그 집단들의 특성, 사회경제적 영향, 예방접종 의무화 혹은 자유화 정책, 윤리 연구 등도 활발하다[4,5,7-10].

기존 연구들은 소아 필수 예방접종에 대한 부모의 태도와 결정에 대해, 세계보건기구가 제시한 예방접종 망설임 (VH, Vaccine Hesitancy)이라는 개념을 흔히 사용한다. 이는 예방접종 서비스를 이용할 수 있음에도 의도적으로 지연하거나 미접종하는 것이다[7]. 이를 기초로 여러 연구들은 네 가지 분류를 하는데, 첫째, 망설임 없이 접종, 둘째, 망설임은 있으나 제 때 접종, 셋째 망설임이 있어서 의도적으로 1개월 이상 지연 접종, 넷째, 망설임으로 인

Correspondence: Soon Young Lee
Department of Preventive Medicine and Public Health, Ajou University School of Medicine, 164 World cup-ro, Yeongtong-gu, Suwon 16499, Korea
E-mail: solee5301@gmail.com

Received: Apr 3, 2019 / Accepted: Jun 15, 2019 / Published: Jul 9, 2019

This article is available from: http://e-epih.org/

This is an open-access article distributed under the terms of the Creative Commons Attribution License (http://creativecommons.org/licenses/by/4.0/), which permits unrestricted use, distribution, and reproduction in any medium, provided the original work is properly cited.

© 2019, Korean Society of Epidemiology

해 의도적으로 접종하지 않음이다[11,12].

한국은 소아 필수 예방접종율이 매우 높다고 알려져 있으며, 예방접종 망설임 연구는 일부만 보고되었다[13,14]. 망설임 개념과 분류도 한국에서는 생소하였다. 그런데 2017년부터 안아키(약 안 쓰고 아이 키우기)가 사회적 이슈가 되면서 예방접종을 포함한 현대의학을 거부하는 움직임이 드러났다. 해외 대안교육 유치원과 학교에 예방접종이나 현대의학 치료를 망설이는 부모들이 있는 것처럼[15], 한국의 대안교육 시설에도 그러한 부모들이 있음을 저자들은 알고 있었다. 물론 전국 실태 조사로 먼저 규모를 파악해야 하지만 현실적 제한이 있었다. 따라서 접근 가능한 부모들을 우선 조사하여 왜 예방접종을 망설이는지 건강믿음모형을 토대로 파악하고자 하였다.

연구방법

예방접종 망설임 부모가 일부 있을 것으로 예상한, 대안교육 유치원과 초등학교에서 대상자를 모집하였다. 대안교육 유치원의 전국 연합회에는 78개소가 가입해 있으며, 그 중 26개소가 경기 남부, 9개소가 경기 북부에 있었다. 연합회에 가입하지 않은 곳은 확인할 수 없었다. 대안교육 학교는 전국적으로 연합회에 246개소(경기 59개소)가 가입해 있으나 대부분 중고등학교이고, 초등학교만 집계한 자료는 없으나 홈페이지가 있는 경우 하나씩 확인하여 약 20곳으로 추정하였다. 표준화된 표본수집을 하기 어려웠고, 다수가 소재한 지역이면서 연구자가 개별 접촉으로 방문할 수 있는 범위가 경기 남부였다. 경기 남부의 유치원 26곳, 초등학교 7곳에 직접 접촉, 우편, 전화, 이메일로 연락을 하였다. 설문에 응한 시설의 대상자 선정 기준은, 2010년 1월 1일 이후 출생한 자녀, 즉 2018년 조사 시점에서 초등학교 2학년 이하 자녀가 한 명 이상 있는 한국인 부모이다. 한국 질병관리본부 어린이 표준예방접종일정표는 만 12세에 예방접종이 완료된다. 따라서 연구 시점 2018년을 기준으로 만 12세 연령, 즉 2007년 출생자부터 조사하려 하였다. 하지만 일정표의 지속적 개정, 만 10-12세 사이에는 예방접종이 추가 2개 밖에 없는 점, 초등학교 입학 후 부모들이 예방접종에 관심이 적어지고 과거 접종 기억이 어려운 점을 고려하여, 대상자 자녀를 2010년 출생자부터로 제한하였다. 그리고 미성년 부모, 한국 국적이 아니거나 귀화한 부모, 조손 양육자는 제외하였다.

저자들이 기존 연구를 토대로 보완한 구조화된 설문지를 사용하였다. 설문은 본인과 자녀의 기본 정보, 건강믿음모형 기반 공통 질문, 예방접종 망설임 네 가지 유형별 문항이다. 건강믿음모형 공통 질문은 개인 인식인 지각한 감수성과 심각성, 조정 요인인 개인적 요인과 행동의로의 계기, 행위 가능성인 지각한 이익과 방해 요인으로서, 기존 연구의 질문을 참조하여 구성하였다[5,11,16,17]. 기존 연구의 질문은 의문문과 서술형인 경우가 모두 있었고, 특정 예방접종을 지목한 경우와 그렇지 않은 경우가 있었다. 저자들은 가급적 문장을 서술형으로 하여 동의 여부를 대답하도록 설계하였으며, 한국 예방접종표를 제시하고 전체 예방접종에 대한 대답을 요청하였다. 또한 최근 연구들이 건강믿음모형에 넣는 자기 효능감 질문을 포함하여[16], 예방접종 네 유형을 결정하는 모형을 구조화하였다(Figure 1). 이에 포함되는 각 문항들은 대답의 동의 여부에 따라 예방접종 망설임 여부를 반영할 수 있도록 한 것이다. 한편, 각 유형별로 예방접종을 망설인 이유와 접종한 이유를 세부적으로 묻는 내용을 기존 연구들을 참조하여 구성하였다[6,18-20]. 문항들은 예방접종을 망설이거나 지연하거나 맞추지 않은 이유, 그리고 영향을 준 사람, 결국 접종한 이유를 세부적으로 묻는 것이다. 기존 연구에서 문항들을 취사선택하고, 한의학, 정부 정책 비협조 시 불이익, 기관 발송 문자 메시지와 같이 한국 특성에 맞는 문항들을 추가하였다. 설문지 원문은 부록으로 첨부하였다. 설문지는 시설에서 직접 배포 후 수거, 대표자나 교사를 통해 전달하여 회수, 혹은 우편 발송 후 회수하였다.

설문 참여자에게는 3색 볼펜 한 자루씩을 제공했고, 다른 보상은 없었다. 통계 분석은 SPSS 23 (IBM Co., Armonk, NY, USA)으로 하였으며, 집단 간 비교는 각 항목별로 교차분석으로 경향성 검정을, 일부 연속변수는 일원배치 분산분석을 하였다. 또한 건강믿음모형에 따른 요인으로 로지스틱 회귀분석을 하였다. 변수 간 공선성 진단은 Cramer의 V값으로 확인하였다. 유의 수준은 0.05이다.

연구결과

2018년 8-11월에 유치원 12곳과 초등학교 2곳에서 설문을 하였고, 시설별로 최소 1명-최대 33명까지 총 141명이 참여하였다. 이 중 2010년생 이후 자녀가 없는 1명, 자녀가 예방접종이 불가능한 의학적 질병이 있는 6명과 미응답 5명, 자녀 동거 여부 미응답 3명을 제외하였다. 중복자 포함 총 12명을 제외하여, 최종 대상자는 129명이었다.

129명은 망설임 없이 접종 43명, 망설였지만 제 때 접종 20명, 1개월 이상 지연 접종 32명, 미접종 34명이었다. 여성 119명, 평균 연령 38.1세(최소 28세-최대 46세), 기혼 125명, 경기도 거주자 127명이었다. 교육 수준은 고졸 3명, 대졸 96명, 대학원졸 31명이었다. 주관적 소득 수준은 상 3명, 중 109명, 하 14명, 미응답 3명이었다. 자녀 수는 1명이 47명, 2명이 69명, 3명이 13명이었다. 각 항목별로 네 집단 간 차이를 비교한 결과 다른 항목들은 유의한 차이가 없었으나, 주관적 소득수준은 의도적 미접종 집단이 소득수준 '하'가 23.5%로 다른 집단보다 많았다($p = 0.006$) (Table 1).

전염병의 위험성 및 예방접종의 이익을 중요하게 생각하는 질문은 지각한 감수성, 지각한 심각성 첫 번째 문항, 지각한 이익, 자기 효능감에 대한 것이다. 네 가지 모두에서 접종 망설임이 없을수

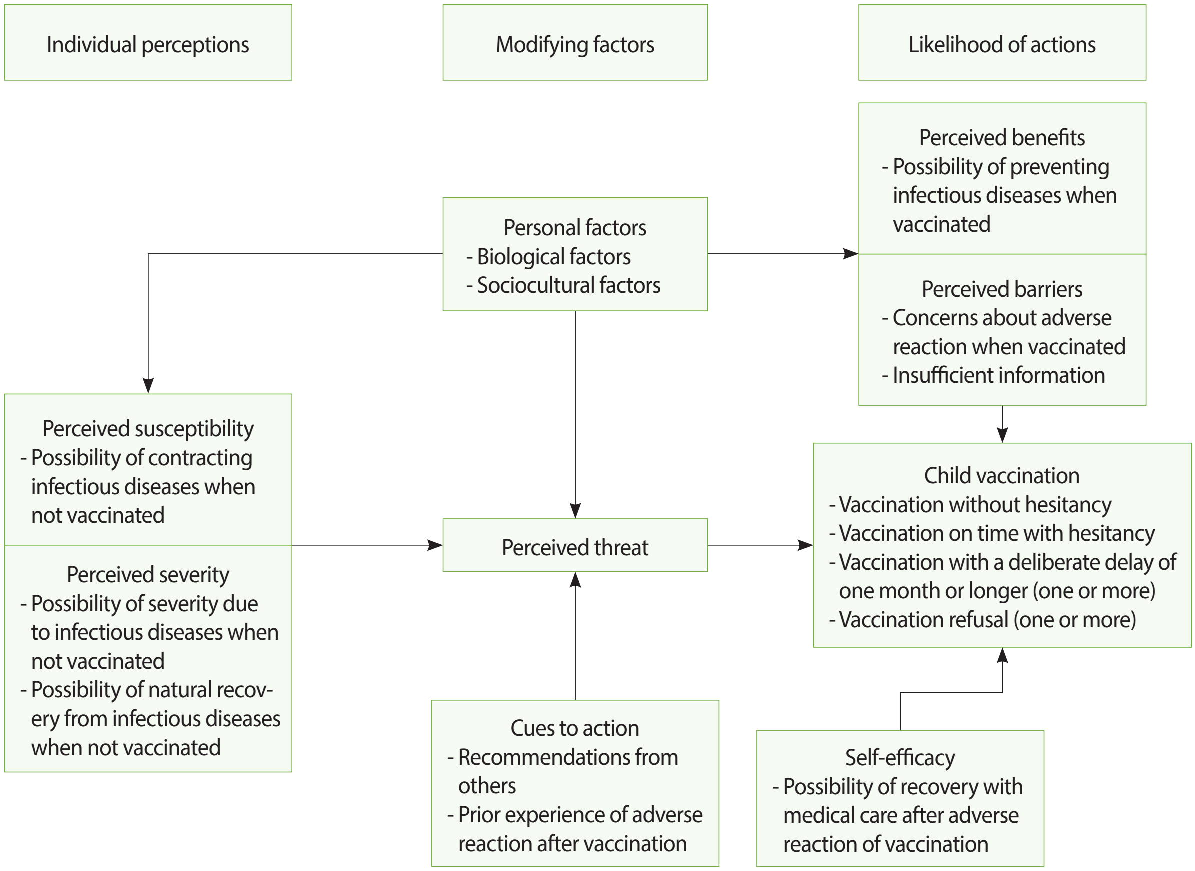

Figure 1. Process of child vaccination decision making according to the Health Belief Model.

록 더 높은 긍정 답변을 하였으며, 통계적으로 유의한 경향성을 보였다. 예방접종 비우호 질문으로 구성한 지각한 방해요인 첫 번째 문항, 행동의로의 계기 두 번째 문항, 지각한 심각성 두 번째 문항에서 모두 의도적 미접종으로 갈수록, 통계적으로 예방접종 부정 경향이 높았다. 지각한 방해요인 두 번째 문항, 행동의로의 계기 첫 번째와 세 번째 문항은 통계적 경향성은 있지만, 미접종 집단이 의도적 지연 접종 집단보다 응답자가 다소 낮아졌다(Table 2).

통계적 차이를 보이는 주관적 소득 수준 및 건강믿음모형의 10개 변수들 간의 상관성을 확인하기 위해 교차분석으로 Cramer의 V값을 확인한 결과, 0.5를 넘는 것은 지각한 감수성과 지각한 심각성의 첫 번째 문항(0.595), 지각한 감수성과 지각한 이익(0.579)이었으며, 0.4를 넘는 것은 지각한 심각성의 첫 번째 문항과 지각한 이익(0.480), 행동의로의 계기 첫 번째와 세 번째 문항(0.411)이었다. 그 외 변수들 간의 Cramer의 V값은 모두 0.4 미만이었다.

Cramer V값 0.5를 기준으로 지각한 감수성 항목을 제외한 건강믿음모형의 9개 문항으로 로지스틱 회귀분석을 실시하였다. 인구사회학적 변수 중 유의한 소득 수준은 상(3명), 중(109명), 하(14명)의 집단별 수 차이가 커서 제외하였다. 이분형 로지스틱 회귀분석을 위해 예방접종 여부는 망설임 없이 접종과 망설였지만 제 때 접종을 합하고, 지연접종 및 미접종 집단을 합했으며, 합쳤을 경우의 각각의 교차분석에서도 유의 수준은 모두 0.05 미만이었다. 조정 전 교차비는 모든 문항에서 통계적으로 유의하였다. 조정 후에는 지각한 심각성($p = 0.042$)이 유의하였다(Table 3).

건강믿음모형에 따른 질문과는 별도로, 예방접종 필요성과 안전성 인식에 대해 네 개 집단 모두에 질문한 결과, 예방접종 망설임이 적을수록 필요성($p < 0.001$)과 안전성($p < 0.001$)에 대한 인식 수준이 높은 통계적 경향성을 확인하였다(Figure 2). 1개월 이상 의도적 지연 혹은 미접종한 예방접종이 있다고 한 사람은 66명이었다. 중복응답을 허용하여, 일본뇌염이 41건으로 가장 많았으며, DTaP (디프테리아, 파상풍, 백일해) 34건, MMR (홍역, 이하선염, 풍진) 34건, IPV (소아마비) 32건, 수두 27건, BCG (결핵) 15건, B형간염 15건 순이었다. 어떤 것인지는 모르지만 접종하지 않은 것이 있음은 20건이었다.

망설였으나 제 때 접종, 1개월 이상 의도적 지연 접종, 의도적 미접종 집단의 망설임 이유를 물었다. 예방접종 부작용 우려나 경험이 모두 60% 이상이었으며, 예방접종 안전관리를 믿을 수 없음도

Table 1. The general characteristics of the parents by their perceptions about vaccinating children

Category	Outcome	No. of people	VH1	VH2	VH3	VH4	p-value[1]
Sex	Female	119	37 (88.1)	19 (95.0)	32 (100)	31 (91.2)	0.398
	Male	9	5 (11.9)	1 (5.0)	0 (0.0)	3 (8.8)	
	No response[2]	1	1	0	0	0	
Age (yr)	Mean±SD	38.1±3.7	37.5±3.6	38.9±4.0	38.5±4.2	37.8±3.2	0.460[3]
	Minimum-Maximum	28-46	30-46	29-44	28-46	32-45	
Marital status	Married	125	41 (95.3)	19 (95.0)	31 (100)	34 (100)	0.158
	Divorced or widowed	3	2 (4.7)	1 (5.0)	0 (0.0)	0 (0.0)	
	No response[2]	1	0	0	1	0	
Education level	Graduated high school	3	1 (2.3)	1 (5.0)	1 (3.1)	0 (0.0)	0.532
	Graduated college or university	95	31 (72.1)	11 (55.0)	26 (81.3)	27 (79.4)	
	Graduated graduate school	31	11 (25.6)	8 (40.0)	5 (15.6)	7 (20.6)	
Subjective income level	Upper	3	3 (7.1)	0 (0.0)	0 (0.0)	0 (0.0)	0.006
	Middle	109	36 (85.7)	19 (95.0)	30 (93.8)	24 (70.6)	
	Lower	14	3 (7.1)	1 (5.0)	2 (6.3)	8 (23.5)	
	No response[2]	3	1	0	0	2	
No. of children	1	47	17 (39.5)	10 (50.0)	8 (25.0)	12 (35.3)	0.355
	2	69	22 (51.2)	9 (45.0)	20 (62.5)	18 (52.9)	
	3	13	4 (9.3)	1 (5.0)	4 (12.5)	4 (11.8)	
Total		129 (100)	43 (33.3)	20 (15.5)	32 (24.8)	34 (26.4)	-

Values are presented as number (%).
VH1, vaccination without hesitancy; VH2, vaccination on time with hesitancy; VH3, vaccination with a deliberate delay of one month or longer (one or more); VH4, vaccination refusal (one or more); SD, standard deviation.
[1]Chi-square for trend test.
[2]No response are not included in the percentage.
[3]Age– analysis of variance.

50% 안팎 혹은 그 이상이었다. 각 집단별로 망설임의 가장 큰 이유는, 망설였으나 제 때 접종 집단은 부작용 우려나 경험, 안전관리 불신, 정부 정책 불신 순이었다. 지연 접종 집단은 부작용 우려나 경험, 정부 정책 불신, 안전관리 불신 순이었다. 미접종 집단은 부작용 우려나 경험, 안전관리 불신, 예방접종 무의미 순이었다(Table 4). 망설임에 영향을 준 사람은, 망설였지만 제 때 접종 집단은 인터넷/블로그/SNS/책/방송 등을 통해 접한 예방접종 반대 활동가, 스스로 결정, 지인 순이었다. 지연 접종 집단은 스스로 결정, 반대 활동가, 지인 및 가족 순이었다. 미접종 집단은 스스로 결정, 반대 활동가, 지인, 소속 단체 활동가, 한의학이나 대체의학 전문가 순이었다(Table 5).

망설임 없이 접종, 망설였으나 제 때 접종, 1개월 이상 의도적 지연 접종 집단에 예방접종을 맞춘 이유를 물었다. 망설임 없이 접종한 집단은 부모 스스로의 판단(72.1%), 병의원이나 보건소 등 기관에서 보내는 안내 문자나 우편물(53.5%), 자녀를 진료한 의사의 설명(48.8%) 순이었다. 망설였으나 제 때 접종한 집단은 부모 스스로의 판단(85.0%), 정부 정책 비협조 시 자녀 불이익 우려(30.0%) 순이었다. 의도적 지연 접종 집단은 부모 스스로의 판단(59.4%), 정부 정책 비협조 시 자녀 불이익 우려(37.5%), 자녀를 진료한 의사의 설명(28.1%) 순이었다.

고 찰

기존 연구에서는 망설임 집단이 교육과 소득 수준이 높은 경향이 있는데[11,21,22], 이번 연구에서 지연 접종, 미접종 집단에서 대졸 이상이 각각 96.9, 100.0%으로 높았다. 하지만 망설임 없이 접종, 망설였지만 제 때 접종 집단도 각각 97.7, 95.0%로 높아서 집단 간 차이가 없었다. 소득 수준은 정량적 측정이 아닌 주관적 응답이었는데, 망설임 없이 접종부터 의도적 지연 접종 집단까지 '중' 대답이 85.7, 95.0, 93.8%로 높았으나, 의도적 미접종 집단은 '중' 79.4%, '하' 23.5%여서 통계적 차이가 있었다. 하지만 모수가 적어서 경향성만 볼 수 있었으며, 그 이유까지 파악할 수는 없었다.

건강믿음모형으로 분석하면, 예방접종 망설임이 적은 집단일수록 미접종 시 전염병에 대한 우려가 높고, 이것이 지각한 위협에 영향을 주어 예방접종을 수용한다. 반대로 망설이는 집단일수록 미접종 시 전염병에 걸려도 자연적 회복 가능성을 높게 생각하며, 이는 지각한 위협을 낮추어 접종을 하지 않는다. 그리고 망설임이 적을수록 예방접종이 전염병을 예방할 수 있다는 이익 인식이 높아서, 접종 결정에 긍정 영향을 준다. 반대로 접종 시 부작용을 우려하거나 접종에 대한 충분한 정보를 제공 받지 못했음은 망설이는 집단에서 더 높게 나타나며, 미접종에 영향을 준다. 다만 정보 불충분은 접종 집단에서도 50%를 상회한다. 행동으로의 계기는

Table 2. Levels of factors of the health belief model by vaccine hesitancy

Questions	Agreed/ respondents	VH1	VH2	VH3	VH4	p-value[1]
Perceived susceptibility<br>If my child does not receive the required vaccinations, she/he could be affected by infectious diseases	79/124 (63.7)	39 (90.7)	16 (80.0)	14 (46.7)	10 (32.3)	<0.001
I don't know[2]	5	0	0	2	3	
Perceived severity<br>If my child does not receive the required vaccinations, she/he could develop a severe condition (example: inpatient treatment at a medical facility) due to infectious diseases	62/121 (51.2)	36 (83.7)	8 (50.0)	11 (36.7)	7 (21.9)	<0.001
I don't know[2]	8	0	4	2	2	
Even if my child does not receive the required vaccinations and is affected by infectious diseases, she/he can sufficiently recover naturally without medical treatment	36/113 (31.9)	6 (14.3)	3 (20.0)	12 (42.9)	15 (53.6)	<0.001
I don't know[2]	16	1	5	4	6	
Perceived benefits<br>If my child receives the required vaccinations, infectious diseases can be effectively prevented	72/117 (61.5)	41 (95.3)	15 (78.9)	12 (44.4)	4 (14.3)	<0.001
I don't know[2]	12	0	1	5	6	
Perceived barriers<br>If my child receives the required vaccinations, adverse reactions might occur	104/120 (86.7)	30 (76.9)	16 (84.2)	28 (93.3)	30 (93.8)	0.024
I don't know[2]	9	4	1	2	2	
I have not received trustable and sufficient information about the required vaccinations for my child	88/126 (69.8)	23 (56.1)	12 (63.2)	27 (84.4)	26 (76.5)	0.019
I don't know[2]	3	2	1	0	0	
Cues to action<br>Have your child actually had an experience of adverse reactions after receiving the required vaccination?	28/129 (21.7)	2 (4.7)	4 (20.0)	11 (34.4)	11 (32.4)	0.001
I don't know[2]	0	0	0	0	0	
Have you directly or indirectly heard of cases in which the child of someone close to you has delayed or refused the required vaccination?	101/129 (78.3)	25 (58.1)	17 (85.0)	28 (87.5)	31 (91.2)	<0.001
I don't know[2]	0	0	0	0	0	
Have you directly or indirectly heard of cases in which the child of someone close to you has experienced adverse reactions after receiving the required vaccination?	52/129 (40.3)	7 (16.3)	10 (50.0)	18 (56.3)	17 (50.0)	0.001
I don't know[2]	0	0	0	0	0	
Self-efficacy<br>Even if my child experiences adverse reactions due to required vaccinations, she/he can recover through sufficient medical treatment	54/102 (52.9)	27 (81.8)	10 (62.5)	9 (34.6)	8 (29.6)	<0.001
I don't know[2]	27	10	4	6	7	
Total	129 (100)	43 (100)	20 (100)	32 (100)	34 (100)	

Values are presented as number (%).
VH1, vaccination without hesitancy, in time; VH2, vaccination with hesitancy, in time; VH3, vaccination with hesitancy, intentionally, over 1 month delayed; VH4, vaccination refusal, intentionally, over one vaccine.
[1]Chi-square for trend test.
[2]Respondents of 'I don't know' are not included in the percentage.

예방접종의 부작용에 대한 직간접적 경험으로서, 이 요인 수준이 높아지면 지각한 위협을 낮추어 미접종에 영향을 주는데, 해당 세 항목 모두 망설임 집단일수록 요인 수준이 높아서 미접종에 영향이 있음을 알 수 있다. 예방접종 부작용 발생 시 충분히 의학적 조치를 통해 완쾌할 수 있다는 자기 효능감 항목은, 망설임 없이 접종 집단일수록 높은 수준이어서, 접종 결정에 긍정 영향이다. 단변량 분석에서 모든 요인이 통계적 유의성을 보여서(Table 2), 건강믿음 모형에 따른 예방접종 망설임 결정 모형의 타당성을 확인하였다. 다변량 분석을 실시하였는데, 연구 대상자 수가 많지 않아서 구조방정식 등 통합 분석이 아닌 로지스틱 회귀분석으로 시행하였다. 그 결과, 지각한 심각성 항목인 미접종 시 전염병에 걸려 심각한 상태에 이를 수 있다는 인식이 접종 수용 요인으로 유의하였다(Table

Table 3. Results of a logistic regression analysis on the factors of vaccine hesitancy

Questions		Unadjusted	Adjusted[1]
Perceived severity			
If my child does not receive the required vaccinations, she/he could develop a severe condition (example: inpatient treatment at a medical facility) due to infectious diseases.	Disagree	1.0 (reference)	1.0 (reference)
	Agree	7.2 (3.2, 16.0)	6.5 (1.1, 38.9)
Even if my child does not receive the required vaccinations and is affected by infectious diseases, she/he can sufficiently recover naturally without medical treatment.	Disagree	1.0 (reference)	1.0 (reference)
	Agree	5.0 (2.1, 12.0)	1.3 (0.2, 7.4)
Perceived benefits			
If my child receives the required vaccinations, infectious diseases can be effectively prevented.	Agree	1.0 (reference)	1.0 (reference)
	Disagree	22.8 (8.2, 63.3)	5.0 (0.9, 28.6)
Perceived barriers			
If my child receives the required vaccinations, adverse reactions might occur.	Disagree	1.0 (reference)	1.0 (reference)
	Agree	3.8 (1.1, 12.5)	2.7 (0.4, 18.1)
I have not received trustable and sufficient information about the required vaccinations for my child.	Disagree	1.0 (reference)	1.0 (reference)
	Agree	2.9 (1.3, 6.4)	1.0 (0.2, 4.8)
Cue to action			
Have your child actually had an experience of adverse reactions after receiving the required vaccination?	No	1.0 (reference)	1.0 (reference)
	Yes	4.8 (1.8, 12.7)	2.0 (0.2, 19.3)
Have you directly or indirectly heard of cases in which the child of someone close to you has delayed or refused the required vaccination?	No	1.0 (reference)	1.0 (reference)
	Yes	4.2 (1.6, 10.8)	4.1 (0.6, 28.0)
Have you directly or indirectly heard of cases in which the child of someone close to you has experienced adverse reactions after receiving the required vaccination?	No	1.0 (reference)	1.0 (reference)
	Yes	3.1 (1.5, 6.4)	3.4 (0.6, 20.0)
Self-efficacy			
Even if my child experiences adverse reactions due to required vaccinations, she/he can recover through sufficient medical treatment.	Agree	1.0 (reference)	1.0 (reference)
	Disagree	6.5 (2.7, 15.6)	1.3 (0.2, 7.4)

Values are presented as odds ratio (95% confidence interval).
[1]Adjusted: Logistic regression model (entered), explanatory power 67.1%, goodness-of-fit test p=0.509.

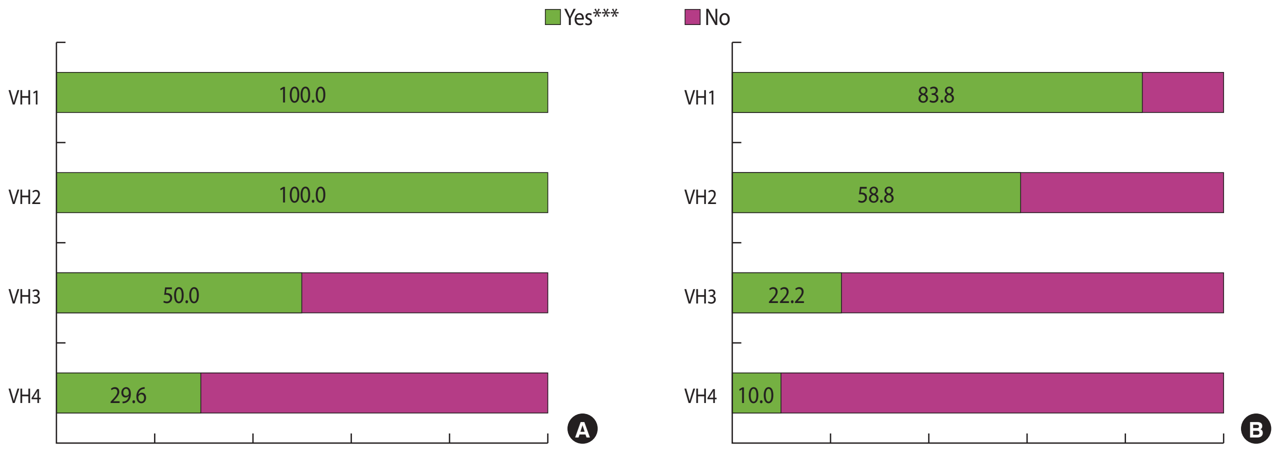

Figure 2. Levels of perception about the (A) necessity and (B) safety of child vaccinations. VH1, vaccination without hesitancy; VH2, vaccination on time with hesitancy; VH3, vaccination with a deliberate delay of one month or longer (one or more); VH4, vaccination refusal (one or more). ***p<0.001 from chi-square for trend test.

3). 이 요인의 집단 간 인식차의 이유를 파악하려면, 대규모 집단 간 비교 연구 또는 미접종 집단에 대한 질적 연구 등이 필요하다.

망설임 집단이 의도적으로 지연하거나 미접종한 예방접종은 일본뇌염, DTaP, MMR, IPV가 조금 더 많았다. DTaP, IPV는 첫 접종은 생후 2개월에 시작하지만 만 4-6세 때까지도 접종이 여러 번 있으며, 일본뇌염, MMR은 12개월 이후 첫 접종을 한다. 반면 출생 직후 바로 접종하는 BCG, B형간염의 미접종 건수가 상대적으로 낮다. 이러한 순위는 부작용에 대한 개인 경험이 이후 미접종에 영향을 주었기 때문으로 추정하는데, 건강믿음모형 분석에서도 이 부분의 항목이 유의하였다. 그리고 망설였지만 제 때 접종,

Table 4. Reasons for vaccine hesitancy

Reasons for vaccine hesitancy	Classification by vaccine hesitancy (responded "yes")[1]		
	VH2	VH3	VH4
Because I do not think that vaccination meaningfully prevents infectious diseases	4 (20.0)	12 (38.7)	20 (58.8)
Because of concerns about the adverse reactions caused by vaccinations or because of prior experiences with small or large adverse reactions	13 (65.0)	22 (68.8)	25 (75.8)
Because I cannot trust the vaccination policies of the government	5 (25.0)	17 (53.1)	18 (54.5)
Because I cannot trust the vaccination safety management of pharmaceutical companies and medical institutions	10 (50.0)	15 (46.9)	22 (68.8)
Because of religious beliefs	0 (0.0)	0 (0.0)	0 (0.0)
Because I trust Korean traditional medicine or alternative medicine more than modern medical science, or because of naturalistic beliefs	3 (15.0)	7 (21.9)	7 (21.2)
Total	20 (100)	32 (100)	43 (100)

Values are presented as number (%).
VH2, vaccination with hesitancy, in time; VH3, vaccination with hesitancy, intentionally, over 1 month delayed; VH4, vaccination refusal, intentionally, over one vaccine.
[1]Multiple responses possible per item.

Table 5. Person(s) who had an impact on vaccine hesitancy

Person(s) who had an impact on the vaccine hesitancy	Classification by vaccine hesitancy (responded with "yes")[1]		
	VH1	VH2	VH3
Family (spouse, parents, male siblings, female siblings, cousins, etc.)	1 (5.0)	11 (34.4)	6 (17.6)
Acquaintances (friends, colleagues, neighbors, etc.)	8 (40.0)	11 (34.4)	18 (52.9)
Leaders or activists of a joined organization (social groups, civil society groups, etc.)	2 (10.0)	6 (18.8)	13 (39.4)
Leaders or activists of a joined organization (religious group)	0 (0.0)	0 (0.0)	0 (0.0)
Anti-vaccination activists whom I found through web, blogs, social network services, and broadcasts	11 (55.0)	12 (37.5)	18 (54.5)
Experts of Korean traditional medicine or alternative medicine	1 (5.0)	8 (25.0)	10 (30.3)
Decision made by myself	10 (50.0)	25 (78.1)	25 (75.8)
Total	20 (100)	32 (100)	43 (100)

VH1, Vaccination without hesitancy, in time; VH2, vaccination with hesitancy, in time; VH3, vaccination with hesitancy, intentionally, over 1 month delayed.
[1]Multiple responses possible per item.

지연 접종, 미접종의 세 집단 모두 예방접종 부작용 우려나 경험이 65.0-75.8%로 망설임의 이유로 가장 높았고[표 4], 안전성에 대한 신뢰도가 낮아지며(Figure 2), 건강믿음모형 요인에서 네 집단 모두 절반 이상이 예방접종 정보가 불충분하다고 대답한 것을 보면, 홍보와 소통의 중요성을 알 수 있다. 또한 예방접종 안전관리와 정부 정책에 대한 불신의 수준도 고려할 때[표 4], 부작용에 대한 정보와 함께 예방접종 정책과 관리 체계 이해도 의사소통에서 중요하다. 이러한 정보를 충분히 제공 받고 전문가와 의사소통을 하는 것은, 접종 결정을 하는 부모들의 권리이고 중요한 요소이다. 이를 위해 어떤 접근법이 유효한지 다양한 해외 연구들이 있지만, 나라마다 사회제도와 문화적 차이로 인해 결과는 다양하다[5,8,10]. 한국에서는 어떤 접근법이 적절한지 연구가 필요하다.

예방접종을 망설이고 미접종하는 이유는, 예방접종 부작용 우려나 경험, 정책 및 안전관리에 대한 불신, 전염병 예방의 의미를 인정하지 않음 등이 주요하였다(Table 4). 망설임 집단이 보건의료 체계를 충분히 신뢰하지 못하고 있음을 추론할 수 있다. 그리고 미접종에 영향을 준 사람은, 온라인 반대 활동가, 시민사회 단체 관계자, 한의사이나 대체의학 전문가였는데 특히 미접종 집단일수록 이들의 영향력이 더 크게 나타났다. 해외 기존 연구에서도 대체의학의 영향이 보고되었다[21]. 다만 한국은 한의학이 제도권 내에 있으므로 한의학과 대체의학을 어떻게 구분하여 연구할지가 향후 과제이다. 또한 기존 연구들과 마찬가지로 온라인으로 접한 예방접종 반대 활동가의 영향도 확인되된다[3,6,22]. 망설임 이슈에 적절하게 대응하기 위해서는, 보건당국과 의료인들은 이러한 사회적 움직임과 관련자들의 영향력을 인지해야 한다[2]. 한편, 민주주의 국가에서 비난, 규제, 처벌 중심의 접근과 시민들을 가르침

과 홍보의 대상으로만 여기는 방식은 효과적이지 않음도 고려해야 한다[22-34]. 이런 여러 측면을 모두 종합하여, 보건관계자들은 예방접종 망설임에 영향을 주는 사람들 및 영향을 받는 부모들과 충분하고 적절한 의사소통을 해야 한다[11,24-26].

예방접종을 망설였지만 결국 수용한 요인은 의사의 설명, 미접종 시 자녀 불이익, 기관의 안내 문자와 우편물 등이었다. 해외 기존 연구도 유사한 결과이지만 의사가 가장 결정적이라는 연구에 비해[11,12], 이번 연구의 망설이다가 접종한 집단에서 그 비율이 30%를 넘지 않았다. 의사의 진료 시간이 짧은 한국 의료환경이 영향을 준 것인지 연구해 볼 필요가 있다. 기존 연구에서는 일차의료 및 의료전달체계가 강한 국가들은 일반주치의가 중요한 역할을 하며, 일차진료로 소아과를 바로 접하는 국가들은 소아과 의사 역할이 중요하다[3,27]. 이러한 점들을 참조하여 한국의 상황 및 향후 방안을 연구해야 한다. 한편, 미접종 시 자녀가 받을 불이익을 우려하여 접종을 결정하는 비중도 높은데, 규제가 접종률 향상에 긍정적일수도 부정적일수도 있다는 연구 결과가 모두 있다[12,28, 29]. 특히 국민 주권주의가 강하고 민주주의 체계가 잘 정착한 국가들에의 연구일수록 규제 강화에는 부정적 경향을 보이기도 한다[9,10,30]. 한국에서도 역사적, 사회적, 문화적 배경을 고려한 연구가 필요하다[31].

일반적으로 예방접종 망설임 연구의 목표는 어떻게 하면 부모들에게 소아 예방접종을 맞추도록 할 것인가이다[1,5,7,22]. 특히 이들이 상당한 규모이면 감염병 유행 파급력을 갖기 때문이다[30, 32]. 하지만 한국에서는 이들을 매우 소수로 추정하기 때문에 유의미한 수준의 감염병 유행 파급력을 갖기는 쉽지 않다[13,14]. 또한 강압적 규제 중심으로는 해결보다는 갈등과 회피 등 문제만 더 커질 수도 있다[3,12,22,33]. 더욱이 이들은 보건의료체계에 대한 신뢰가 낮다는 연구들이 알려져 있어서 규제 중심 정책은 신중해야 한다[34]. 망설임이 있는 부모들과 포괄적 신뢰를 쌓는 의사소통을 하여, 사회적 합의가 가능한 해결 방안을 찾아야 한다[2,35].

그동안 예방접종 망설임 연구는 주로 서구권과 일본을 중심으로 보고되었고, 한국의 실태 조사나 연구는 미흡하였다. 저자들의 연구도 전국적 실태 파악이 아니며, 특정 집단에서 소수의 부모를 대상으로 하여 대표성에 한계가 있다. 하지만 예방접종 망설임 부모들이 집중되어 있을 가능성이 있는 대안교육 유치원과 초등학교를 대상으로 하였기에, 차후 필요한 대규모 연구나 질적 연구에 참조가 될 것이다. 특히 대표성과 대상자 수의 한계를 보완하기 위해, 건강믿음모형을 적용하여 예방접종 긍정 및 부정 이유들이 접종 여부에 어떻게 연결되는지 논리적으로 파악하여 한계점을 보완하였다. 그 결과 해외 기존 연구들과 유사하게, 예방접종 망설임 요인들이 작용하고 있음을 알 수 있었다. 만약 실태 조사를 통해 다른 선진국들과 마찬가지로 상당한 규모의 망설임 집단이 있고 영향력 확장 가능성이 있다면, 이번 연구 결과는 추후 연구 및 정책 결정에 중요한 참조가 될 것이다. 한편, 한국은 공식적 한의사 제도, 일차의료체계가 미흡하고 진료 시간이 짧은 특성, 미접종 시 자녀 불이익 등의 특성들이 있다. 향후 한국적 특성에 초점을 맞춘 추가 연구들이 특히 중요하다.

감사의 글

건강믿음모형을 반영하여 설문 문항을 구성하는데 도움을 준 아주대학교 보건대학원 이자경 자문교수에게 깊은 감사의 말씀을 드린다.

참고문헌

1. Edwards KM, Hackell JM; Committee on Infectious Diseases, & Committee on Practice and Ambulatory Medicine. Countering vaccine hesitancy. Pediatrics 2016;138:e20162146.
2. Blume S. Anti-vaccination movements and their interpretations. Soc Sci Med 2006;62:628-642.
3. McIntosh ED, Janda J, Ehrich JH, Pettoello-Mantovani M, Somekh E. Vaccine hesitancy and refusal. J Pediatr 2016;175:248-249.
4. Lo NC, Hotez PJ. Public health and economic consequences of vaccine hesitancy for measles in the United States. JAMA Pediatr 2017;171:887-892.
5. Tsuchiya Y, Shida N, Izumi S, Ogasawara M, Kakinuma W, Tsujiuchi T, et al. Factors associated with mothers not vaccinating their children against mumps in Japan. Public Health 2016;137:95-105.
6. Sandhofer MJ, Robak O, Frank H, Kulnig J. Vaccine hesitancy in Austria: a cross-sectional survey. Wien Klin Wochenschr 2017; 129:59-64.
7. World Health Organization. Report of the SAGE working group on vaccine hesitancy; 2014 [cited 2019 Jul 8]. Available from: https://www.who.int/immunization/sage/meetings/2014/october/SAGE_working_group_revised_report_vaccine_hesitancy.pdf.
8. Di Pietro ML, Poscia A, Teleman AA, Maged D, Ricciardi W. Vaccine hesitancy: parental, professional and public responsibility. Ann Ist Super Sanita 2017;53:157-162.
9. Clarke S, Giubilini A, Walker MJ. Conscientious objection to vaccination. Bioethics 2017;31:155-161.
10. Parasidis E, Opel DJ. Parental refusal of childhood vaccines and medical neglect laws. Am J Public Health 2017;107:68-71.
11. Chung Y, Schamel J, Fisher A, Frew PM. Influences on immunization decision-making among US parents of young children. Matern Child Health J 2017;21:2178-2187.
12. McClure CC, Cataldi JR, O'Leary ST. Vaccine hesitancy: where we are and where we are going. Clin Ther 2017;39:1550-1562.
13. Larson HJ, de Figueiredo A, Xiahong Z, Schulz WS, Verger P, Johnston IG, et al. The state of vaccine confidence 2016: global insights through a 67-country survey. EBioMedicine 2016;12:295-301.
14. Park B, Choi EJ, Park B, Han H, Cho SJ, Choi HJ, et al. Factors influencing vaccination in Korea: findings from focus group interviews. J Prev Med Public Health 2018;51:173-180.
15. Brennan JM, Bednarczyk RA, Richards JL, Allen KE, Warraich GJ, Omer SB. Trends in personal belief exemption rates among alternative private schools: Waldorf, Montessori, and holistic kindergartens in California, 2000–2014. Am J Public Health 2017;107: 108-112.

16. Hayden JA. Introduction to health behavior theory. 2nd ed. Sudbury: Jones & Bartlett Learning; 2013, p. 63-71.
17. Donadiki EM, Jiménez-García R, Hernández-Barrera V, Sourtzi P, Carrasco-Garrido P, López de Andrés A, et al. Health Belief Model applied to non-compliance with HPV vaccine among female university students. Public Health 2014;128:268-273.
18. Smith PJ, Humiston SG, Marcuse EK, Zhao Z, Dorell CG, Howes C, et al. Parental delay or refusal of vaccine doses, childhood vaccination coverage at 24 months of age, and the Health Belief Model. Public Health Rep 2011;126 Suppl 2:135-146.
19. Carrion ML. An ounce of prevention: identifying cues to (in) action for maternal vaccine refusal. Qual Health Res 2018;28:2183-2194.
20. Smith LE, Weinman J, Amlôt R, Yiend J, Rubin GJ. Parental expectation of side effects following vaccination is self-fulfilling: a prospective cohort study. Ann Behav Med 2019;53:267-282.
21. Bryden GM, Browne M, Rockloff M, Unsworth C. Anti-vaccination and pro-CAM attitudes both reflect magical beliefs about health. Vaccine 2018;36:1227-1234.
22. Jung M. Challenges of vaccinations in the era of new media communication. Health Care Manag (Frederick) 2018;37:142-146.
23. Carpiano RM, Fitz NS. Public attitudes toward child undervaccination: a randomized experiment on evaluations, stigmatizing orientations, and support for policies. Soc Sci Med 2017;185:127-136.
24. Brelsford D, Knutzen E, Neher JO, Safranek S. Clinical inquiries: which interventions are effective in managing parental vaccine refusal? J Fam Pract 2017;66:E12-E14.
25. Bocquier A, Fressard L, Cortaredona S, Zaytseva A, Ward J, Gautier A, et al. Social differentiation of vaccine hesitancy among French parents and the mediating role of trust and commitment to health: a nationwide cross-sectional study. Vaccine 2018;36: 7666-7673.
26. McKee C, Bohannon K. Exploring the reasons behind parental refusal of vaccines. J Pediatr Pharmacol Ther 2016;21:104-109.
27. Cordrey K, McLaughlin L, Das P, Milanaik R. Pediatric resident education and preparedness regarding vaccine-preventable diseases. Clin Pediatr (Phila) 2018;57:327-334.
28. Marshall GS, O'Leary ST. Dismissal policies for vaccine refusal. JAMA Pediatr 2018;172:1101.
29. Lee S, Riley-Behringer M, Rose JC, Meropol SB, Lazebnik R. Parental vaccine acceptance: a logistic regression model using previsit decisions. Clin Pediatr (Phila) 2017;56:716-722.
30. Lee C, Whetten K, Omer S, Pan W, Salmon D. Hurdles to herd immunity: distrust of government and vaccine refusal in the US, 2002-2003. Vaccine 2016;34:3972-3978.
31. Attwell K, Navin MC, Lopalco PL, Jestin C, Reiter S, Omer SB. Recent vaccine mandates in the United States, Europe and Australia: a comparative study. Vaccine 2018;36:7377-7384.
32. Rosen JB, Arciuolo RJ, Khawja AM, Fu J, Giancotti FR, Zucker JR. Public health consequences of a 2013 measles outbreak in New York City. JAMA Pediatr 2018;172:811-817.
33. Colgrove J. Vaccine refusal revisited—the limits of public health persuasion and coercion. N Engl J Med 2016;375:1316-1317.
34. Rozbroj T, Lyons A, Lucke J. Psychosocial and demographic characteristics relating to vaccine attitudes in Australia. Patient Educ Couns 2019; 102:172-179.
35. Larson HJ. Vaccine trust and the limits of information. Science 2016; 353:1207-1208.
